# Biocompatible Self-Healing Hydrogel for VAT 3D Printing

**DOI:** 10.1021/acsmaterialsau.5c00194

**Published:** 2026-02-13

**Authors:** Maria D’Aloia, Désirée Baruffaldi, Sandra Dirè, Emanuela Callone, Angelo Angelini, Candido Fabrizio Pirri, Ignazio Roppolo, Francesca Frascella

**Affiliations:** † Department of Applied Science and Technology, 19032 Politecnico di Torino, Corso Duca degli Abruzzi 24, Turin 10129, Italy; ‡ Department of Chemistry, Biology and Biotechnology, University of Perugia, Via Elce di Sotto 8, Perugia 06123, Italy; § PoliTOBioMEDLab, 19032 Politecnico di Torino, Corso Duca degli Abruzzi 24, Torino 10129, Italy; ∥ ”Klaus Müller” Magnetic Resonance Lab, Department of Industrial Engineering, 387464 University of Trento, Via Sommarive 9, Trento 38123, Italy; ⊥ Italian National Institute of Metrological Research (INRiM), Strada delle Cacce, 91, Turin 10135, Italy; # Center for Sustainable Future Technologies, Italian Institute of Technology, Via Livorno 60, Turin 10144, Italy

**Keywords:** self-healing, hydrogel, VAT 3D printing, biocompatible, borate-ester

## Abstract

Self-healing hydrogels (SHHs) are promising materials in tissue engineering due to their ability to mimic the biomechanical properties of biological tissues and autonomously repair damage and can serve as ideal three-dimensional scaffolds for cell proliferation and differentiation. However, conventional processing methods, such as extrusion-based printing, often limit the complexity and resolution of the fabricated structures. VAT photopolymerization, particularly digital light processing (DLP), on the other hand, offers advantages in terms of resolution, printing speed, and design freedom, making it an attractive approach for developing SHHs. This study aims to develop a biocompatible and self-healing hydrogel through DLP, enhancing the structural complexity while maintaining self-repairing properties. The hydrogel is made from polyethylene glycol diacrylate (PEGDA), hydroxyethyl methacrylate (HEMA), dithiothreitol (DTT), and borax, and the solvent is PBS. Cross-linking occurs by radical photopolymerization, in which PEGDA and HEMA serve as cross-linkable monomers, while DTT and borax form borate–ester bonds, imparting self-repairing properties. Complex 3D structures were fabricated by using a commercial DLP printer, and self-healing properties were assessed. Chemical (FTIR, NMR), rheological, and mechanical analyses were performed along with cytocompatibility tests. The hydrogel exhibited successful 3D printability, allowing the fabrication of complex structures. Self-healing tests demonstrated that, after 72 h, the samples could self-repair and withstand tensile forces, maintaining their integrity after multiple damage–repair cycles. Chemical and mechanical characterization confirmed the stability and viscoelastic behavior of the material, while preliminary cytocompatibility assays indicated the suitability for tissue engineering applications. DLP-based printing enables the fabrication of self-healing hydrogels with improved resolution and design freedom. The developed hydrogel exhibits promising mechanical properties and biocompatibility, making it a strong candidate for tissue engineering applications.

## Graphical abstract

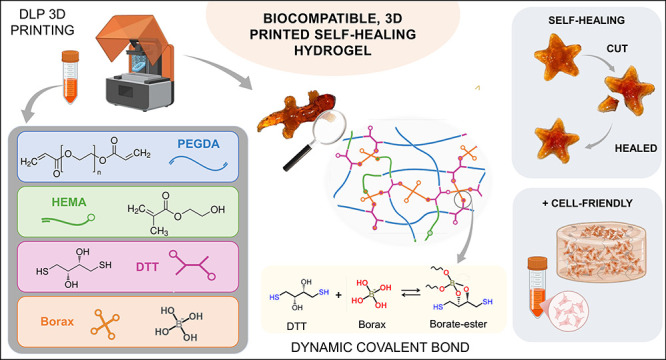

## Introduction

Hydrogels are soft three-dimensional networks consisting of hydrophilic polymers with the ability to absorb a large amount of water, often employed to mimic living tissues,^1–3^ thanks to their soft, hydrophilic nature and tunable stiffness.^3,4^ Depending on the materials and design strategies used, hydrogels can have a wide range of physicochemical properties^3^ and can be biomimetic and biocompatible.^1^

Furthermore, many other properties of hydrogels were exploited for biomedical applications^3,5^ such as wound healing,^6,7^ tissue engineering^8,9^ and drug delivery.^4,10,11^ On the other hand, similarly to many living tissues, hydrogels may suffer poor mechanical properties and a high tendency to be damaged or broken.^1,2,12^ For this reason, many investigations in recent years have been focused on the development of the so-called self-healing hydrogels (SHHs), i.e., hydrogels that have the intrinsic ability to self-repair when they undergo damage,^1^ partially or completely restoring the initial properties.^1,13^ SHHs are thus characterized by improved durability, reliability, and safety of the material by avoiding failures.^1^ Additionally, SHHs can also better resemble living tissue dynamics than standard hydrogels owing to their spontaneous healing, similar to natural tissues^1,14^

Generally, SHHs are divided into two categories based on their self-repair mechanisms, which can be either extrinsic or intrinsic.^1,13^ The first exploits the presence of reservoirs of unreacted monomers, which are incorporated into the network: in the case of damage, when the crack reaches these reservoirs, the unreacted monomers are released and function as sealants. This mechanism is particularly effective in restoring large portions of material but can only be achieved once per site.^13^ Alternatively, the intrinsic mechanism is based on the presence of functional groups that can establish new bonds in the case of damage. This allows multiple repairs of the same site, although it is usually effective for small and limited areas of damage.^13^ Intrinsic strategies are generally versatile and result in more stable materials, since their slower cleavage and bond formation kinetics,^13^ which is why they are the most studied and used, especially in biomedical applications. The intrinsic mechanism can be achieved by exploiting the presence of dynamic covalent bonds (chemical cross-linking) or noncovalent supramolecular interactions (physical cross-linking).^1,13^ Examples of noncovalent bonding include hydrogen bonding,^15,16^ host–guest interactions,^17–19^ hydrophobic bonding,^20–22^ and ionic bonding.^23,24^ Dynamic covalent bonding includes imine bonds,^25,26^ Diels–Alder reactions,^27^ acylhydrazone bonds,^28,29^ disulfide bonds,^30^ borate ester bonds,^3,31–33^ oxime bonds,^34^ and others.

In general, the intrinsic self-healing process depends on the dynamic equilibrium of bonds^1,35^ and macromolecular interdiffusion, which occurs spontaneously within a mobile phase;^1,13,35,36^ the movement of polymer chains depends on their structure, molecular weight, temperature, and the possible presence of other solvents.^13,37^ Therefore, unlike extrinsic mechanisms that need no stimulus other than the damage itself to activate the self-repair process, intrinsic mechanisms may need an external stimulus that promotes the mobility of polymer chains,^13,38^ such as temperature, pH, light, or ion concentration.^13,39,40^

Although self-healing mechanisms induced by external stimuli such as light or heat have proven successful,^39,41^ self-healing without external stimuli remains more desirable, especially in the biomedical field,^36,39,42^ where the application of a stimulus might be complicated or harmful.^43^ In hydrogels, the high-water content allows for better mobility of polymer chains by increasing the free volume and distance between molecules. This mobility is beneficial for self-healing systems, as it promotes the reformation of dynamic bonds. However, the increase in chain mobility is often associated with lower mechanical strength and integrity, which strongly depends on the cross-linking density and, therefore, is inversely proportional to self-repair capabilities. Increasing the strength or the amount of cross-links in the network can help improve mechanical performance, but can inhibit self-healing by limiting chain mobility.^13^ Therefore, a balance between sustaining mechanical integrity and allowing for sufficient healing efficiency is crucial in the design of SHHs.

Hydrogels capable of self-healing have been extensively explored in various biomedical applications, with large investigations on bioscaffolds,^44–48^ wound-healing patches,^49–51^ and drug-delivery platforms, among others, but also in reusable and wearable sensors,^52–55^ bioelectronics,^56,57^ and soft actuators.^58–60^ Some dedicated reviews may be further consulted for a more complete picture concerning the different applications of self-healing hydrogels.^13,61–63^

Most classical hydrogels are mechanically weak and prone to damage due to their high water content and soft cross-linked nature. Their polymeric network, most frequently held together by irreversible covalent bonds or loose physical interactions, prevents structural rearrangement or healing after deformation. This lack of healing ability greatly limits their durability and functionality in dynamic biological environments.^64^ These drawbacks, together with the limited control over microstructure and geometry offered by conventional fabrication methods such as extrusion or one-pot synthesis,^65^ have limited the use of hydrogels to in vitro models or low-complexity systems, excluding their full potential in clinically relevant models. Therefore, recent advances have focused on the incorporation of dynamic and reversible chemistries that can promote self-healing ability in the material, together with an adaptive mechanical behavior;^13,65^ at the same time, advances in additive manufacturing technology have enabled the production of hydrogels with an unprecedented geometrical precision and spatial control.

Based on this background, in this work, borate ester bonds, between borate anions and diols, were exploited to fabricate SHHs, since this covalent bond is capable of spontaneously reforming when broken,^3,66–68^ without the need for external stimuli. A scheme of borate-ester formation is shown in Figure 1a. Furthermore, in view of its application in the biomedical field, previous studies showed that borate compounds present no particular toxicity compared to the other organic compounds.^3,69^ The formation of borate–ester bonds is a chemical equilibrium process that can be influenced by environmental factors such as temperature, pH, and the concentration of saccharides such as glucose and fructose, endowing hydrogels with interesting stimuli-responsive behaviors.^3,31,70^ For example, cross-link formation is exothermic (Δ*H* ≈ 1–2 kJ mol^–1^) and so can be inhibited or reversed by heating,^33,71^ while gelation typically occurs in mildly basic conditions (pH∼8–9), close to the p*K*_a_ of the boron compound.^33,72^

**Figure 1:**
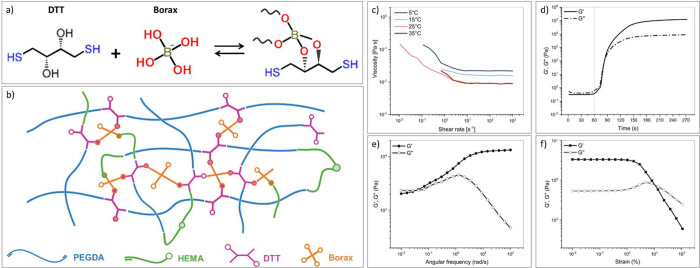
(a) Schematic representation of the reversible bond between DTT and borax to form borate–ester bonds; (b) schematic representation of hydrogel’s network; (c) flow curve of the liquid formulation measured at different temperatures; (d) photorheology measurement; (e) frequency sweep on the gelled samples; and (f) amplitude sweep on the gelled samples. All tests were conducted at room temperature unless specified.

Importantly, the borate-ester system presented in this work represents a highly versatile and modular self-healing mechanism: it can be easily incorporated into a variety of polymer networks, both synthetic and natural, without complex chemical modifications. Therefore, this is an attractive and broadly applicable strategy for the development of adaptive, self-repairing soft materials, particularly in applications where structural integrity and long-term stability are essential.

Traditional preparation of hydrogels usually involves physical/chemical cross-linking, leading to the fabrication of objects with relatively simple geometries, while more complex shapes usually need a mold.^73^ Different methods have been used to fabricate hydrogels (emulsification,^74,75^ lyophilization,^76,77^ electrospinning,^78,79^ photolithography,^80,81^ microfluidics^82–84^ and micromolding^85,86^ ), allowing for the control of hydrogels’ morphology, porosity, and mechanical properties. Even though these traditional techniques are functional, they often suffer from limitations in terms of geometric precision, reproducibility, and scale-up.^87^ The advent of additive manufacturing, particularly three-dimensional (3D) printing, has allowed for layer-by-layer construction of hydrogels with complex and detailed structures in a short time, reproducing the complexity of living tissues. Hydrogels obtained by 3D printing also exhibit superior mechanical properties, making them better suited to meet specific requirements than those prepared by conventional approaches.^73,88–90^

However, 3D printing of SHHs requires more stringent requirements than other hydrogels, as it is critical to balance competing elements, such as water content, cross-linking density, and the effectiveness of chain interactions. For instance, fewer cross-linking points generate softer hydrogels, which facilitate chain mobility but produce structures with poor shape fidelity. On the other hand, high cross-linking density helps shape retention and printing speed, but loses restorative capacity.^13,91^

Extrusion printing, or direct ink writing (DIW), is the most common method of printing hydrogel constructs. In this process, a prepolymer liquid or ink is propelled through a nozzle by pneumatic, mechanical, or screw-driven pressure, and the filaments are patterned in a controlled manner. The extruded material solidifies by gelation, cross-linking, or cooling to form a stable 3D object.^65^ Even if widely used for hydrogel fabrication, extrusion printing is characterized by low resolution (typically 100–400 μm),^87^ limited design, viscosity constraints, and, if a bioink needs to be used, potential cell damage if applied to bioprinting. High pressure required for extrusion can cause cell damage.^87,92^

In contrast to extrusion systems, VAT light-induced 3D printing technologies like Digital Light Processing (DLP) possess significantly higher resolution, greater architectural freedom, as well as higher fabrication speed.^93^ These technologies are based on the photopolymerization of light-sensitive resins that, upon exposure to light, typically ultraviolet (UV) or visible light, undergo spatially controlled cross-linking reactions.^93^ Despite these advantages, self-healing hydrogels compatible with DLP remain limited, creating a materials-design challenge that this work aims to address.

Even though borax-mediated self-healing and its biocompatibility have been previously explored,^3,43,61^ its integration into light-based 3D-printable hydrogel systems remains limited. Existing studies predominantly rely on bulk hydrogels^43,94^ or extrusion-based systems,^95^ which lack fine structural control and exhibit lower resolution compared to light-based 3D printing techniques. Therefore, both the combination of borate ester chemistry with nontoxic, DLP-compatible monomers and the systematic evaluation of printability, mechanical properties, and autonomous healing in printed constructs represent an underexplored area, and this work aims to fill this gap.

Summarizing, in this work, we exploited Digital Light Processing (DLP) as a 3D printing technique, because of its cost-effectiveness, printing speed, and resolution,^96,97^ for the production of SHHs. The developed SHH is based on poly­(ethylene glycol)­diacrylate (PEGDA) and hydroxyethyl methacrylate (HEMA), used as structural and photosensitive components, together with dithiothreitol (DTT) and disodium tetraborate decahydrate (borax), which will form the borate–ester bonds responsible for the self-healing ability. A schematic representation of the hydrogel’s network is reported in **Errore. L’origine riferimento non è stata trovata.**b. Phosphate buffer solution (PBS) (pH between 7 and 7.4) was chosen as the liquid phase. The developed materials were fully characterized and employed for DLP 3D printing, showing excellent restoring properties and promising biocompatibility, crucial aspects for future biological applications.

The ultimate goal of this work is to develop and characterize a DLP-printable self-healing hydrogel based on PEGDA/HEMA/DTT/borax, showing (i) its ability to maintain autonomous, stimulus-free healing, (ii) its compatibility with high-resolution VAT photopolymerization, and (iii) its potential application in biomedical contexts, where both structural fidelity and regenerative capacity play crucial roles. The integration of borate ester dynamics into a photopolymerizable, biocompatible resin represents the key novel contribution of this study.

## Materials and Methods

### Materials

The material selection is explained in detail in the paragraph “formulation design” in the Supporting Information file. PEGDA_700_ (Mn = 700 g/mol), HEMA (99%, Mn = 130.14 g/mol), DTT (97%, Mn = 154.25 g/mol), LAP (>95%), borax (Mn = 381.23 g/mol), and PBS were purchased from Merck Company (Darmstadt, Germany) and used as received. Grapeseed extract (Extratan Vinacciolo, EV) was kindly provided by Oenoitalia Biotecnologie and used as received. Chemical structures of PEGDA, HEMA, DTT, borax, and LAP, together with the LAP absorbance spectrum, are reported in Figure S1.

### Resin Preparation

Preliminary studies were conducted to define the most promising formulation, which has the composition shown in Table 1. More details can be found in the Supporting Information. DTT was added to a 0.1 M solution of borax in PBS and mixed until completely dissolved. In another vial, HEMA, PEGDA, and LAP were dissolved in the remaining weight of PBS, so that the total amount of PBS in the hydrogel is 80% in weight. The contents of the two vials are then combined and gently shaken manually. Finally, grapeseed extract is added and dissolved using a magnetic stirrer (it can take up to 24 h for complete dissolution). The resulting resin is stored at room temperature in a dark vial until needed.

**Table 1:** Composition of the Liquid Formulation Expressed in Absolute Quantities (mg) and Corresponding Weight Percentages Normalized to 1 g of the Total Formulation^a^

component	Quantity (mg)	Wt %	notes
PBS	800	79.5	80 wt % target
borax	22.2	2.2	borax:DTT = 1:3
DTT	66.7	6.6	DTT:acrylates = 1:2
HEMA	44.4	4.4	HEMA:PEGDA_700_ = 1:2
PEGDA_700_	88.9	8.8	HEMA:PEGDA_700_ = 1:2
LAP	1.33	0.13	1% based on acrylates weight (HEMA + PEGDA_700_)
radical scavenger (EV)	5	0.5	0.5% based on total weight excluding LAP

a PBS constitutes the majority phase (∼80 wt %), while the organic reactive components (DTT, HEMA, PEGDA700) are added in defined stoichiometric ratios. The formulation includes LAP (1 wt % relative to acrylates) as a photoinitiator and grapeseed extract (0.5 wt % relative to the total weight excluding LAP) as a radical scavenger. Quantities are adjusted to maintain the specified molar ratios among components.

### Rheological Measurement

Rheological measurements were performed using an Anton PAAR Modular Compact Rheometer (Physica MCR 302, Graz, Austria) in the parallel-plate mode (25 mm diameter). The gap between plates was set to 0.5 mm. Rheological measurements were performed to characterize the liquid formulation and the gelled samples. First, the viscosity of the liquid formulation was evaluated with continuous flow measurements performed with a range of shear rates from 0.01 to 1000 s^–1^. Different temperatures were tested (5, 10, 25, and 35 °C). 3–5 min was given for conditioning the sample to the chosen temperature. A temperature ramp test was conducted with a rate of 2 °C/min, from 4 to 36 °C, maintaining a constant shear rate of 100 s^–1^.

Real-time photorheological measurements were performed to assess the change in viscosity during the photopolymerization process: the UV-light source was provided by positioning the light guide of the UV Hamamatsu LC8 lamp (emission at 365 nm) under the bottom plate. During the measurements, the sample was kept under a constant shear frequency of 1 Hz. The irradiation light was switched on after 60 s to allow the system to stabilize before the onset of polymerization. According to the preliminary amplitude sweep measurements, all of the tests were carried out in the linear viscoelastic region at a strain amplitude of 50%. Amplitude sweep tests were performed in the range of 1 to 1000% strain, with a frequency of 1 Hz. The photorheology was studied as a function of the changes in the shear modulus (G′) and loss modulus (G″) of the sample versus the exposure time. The network parameters cross-link density (ν_e_) was calculated by eq 1:^98^

$$v_{e}=\frac{G^{\prime }N_{A}}{RT}$$ 1

where *R* is the universal gas constant, *T* is the temperature, and *N*_A_ is Avogadro’s number.

A frequency sweep test was performed on the liquid formulation between 0.1 and 100 Hz (0.6–600 rad/s), maintaining a constant strain of 50%, according to the results of the amplitude sweep.

Finally, frequency sweep and amplitude sweep tests were also conducted on the gelled samples. A frequency sweep was conducted between 0.1 and 100 rad/s, maintaining a constant strain of 1% and a temperature of 25 °C. Amplitude sweep was conducted at different constant temperatures (5, 25, and 37 °C) between 0.01 and 1000% strain, maintaining a constant frequency of 1 Hz.

All tests, unless specified, were conducted at 25 °C. Outlier values have been neglected.

### NMR Spectroscopy

Solid-state NMR analyses were carried out with a Bruker 400WB spectrometer operating at a proton frequency of 400.13 MHz. NMR spectra were acquired with a single pulse sequence under the following conditions: 13C frequency: 100.48 MHz, π/2 pulse 4.4 μs, decoupling length 5.9 μs, recycle delay: 3 s, 2k scans.^11^ B frequency: 128.38 MHz, short excitation pulse 1.5 μs, decoupling length 6.6 μs, recycle delay: 5 s, 5 k scans, background subtraction. Samples were packed in a 4 mm zirconia rotor and spun at 8 kHz under air flow. Adamantane and solid NaBH_4_ were used as an external secondary reference. Some experiments were repeated on wet samples (20 μl of H_2_O was added to 80 mg of sample) in order to enhance spectra resolution.

### FTIR Spectroscopy

The IR spectra were recorded with a PerkinElmer Spectrum Two spectrometer in the ATR mode, with a resolution of 4 cm^–1^, averaging 32 scans for each spectrum, in a wavenumber range of 550–4000 cm^–1^. The obtained spectra were referenced at Abs_4000_ = 0.

### 3D Printing

DLP 3D Printing was carried out using a commercial Asiga MAX X27 UV DLP 3D printer with a 385 nm LED light source of 385 nm. The printer has a nominal resolution of 27 × 27 μm^2^ on the xy plane and a build volume of 51.8 × 29.2 × 75 mm^3^. After printing, the excess resin was drained from the parts, and the structures were postcured for 5′ using an Asiga Flash UV chamber. The printing parameters were optimized, and the structures were printed using an LED light intensity of 55 mW/cm^2^ per layer of thickness 70 μm. The first 3 layers are irradiated for 45 s, and the remaining layers for 35 s.

### Self-Healing Behavior

Cuboid specimens were 3D-printed, cut into two halves using a cutter, and kept in contact with each other for different times. The self-healing behavior was observed at different time steps, evaluating the visibility of the cut interface, the hydrogel’s capacity to sustain its own weight, and the capacity to undergo tensile stress without breaking. To determine the minimum time required for effective healing, preliminary tests were carried out on rectangular 3D-printed samples (20 × 7.5 × 2 mm), in which half was stained with a dye (Brilliant Green (BG), Sigma-Aldrich, Merck Company, Darmstadt, Germany), while the other remained unstained. In addition to the aforementioned parameters, dye diffusion across the cut interface was qualitatively monitored as an indicator of molecular mobility and interfacial integration. Samples were observed for 24, 48, and 72 h.

Finally, the self-healing capability was further evaluated through scanning electron microscopy and tensile tests, as described in the following sections.

### Scanning Electron Microscopy

Scanning electron microscopy (SEM) analysis was carried out on hydrogel samples fabricated by Digital Light Processing (DLP) 3D printing. A pair of samples (as-printed and after self-healing) was analyzed in the hydrated state. The electron microscope images were acquired by a FEI Inspect-F field emission gun scanning electron microscope (FEG-SEM) using an Everhart-Thornley secondary electron detector (ETD), using an acceleration voltage of 30 kV.

### Mechanical Analysis

For both tensile and compression tests, a Z3 tensile tester (AML Instruments) with a 500 N load cell equipped with tensile and compression grippers was used. The data were generated and collected using THSSD software. Compression tests were conducted at a rate of 3 mm/min on cylindrical specimens (10 mm diameter and 5 mm thickness). Tensile tests were conducted at a rate of 5 mm/min on dog-bone-shaped specimens (4 cm long, 4 mm thick, 13 mm maximum width, and 6 mm minimum width). The tensile specimens were brought to failure. Then the two parts were brought into contact to allow the specimen to heal. After 72 h, the same specimens were again tested. This procedure was repeated 3 times to evaluate the difference in mechanical properties after self-healing. For each self-healing cycle, control samples, prepared simultaneously with the test samples but not subjected to mechanical failure, were also tested in order to distinguish the effects of damage versus natural aging. Besides, separate groups of samples were soaked in water for various times (1, 7, and 14 days) to examine the effect of high-humidity environments on the mechanical properties. Certain samples were intentionally broken after 1 day of swelling, allowed to heal spontaneously for 1 week at room temperature, and subsequently retested. In both compression and tensile tests, properties such as the elastic modulus, stress at break, and elongation at break were evaluated as the average of values obtained on at least three specimens.

### Biological Tests

#### Sterilization Protocol

Liquid formulation was sterilized according to the following procedure: the resin was heated at 37 °C for 30 min to decrease its viscosity; then, it was filtered through 0.45 and 0.22 μm filters, sequentially. The sterilized resin was kept in the dark at room temperature (RT) until it was needed.

#### Viability Assessment

Human lung adenocarcinoma cell lines, A549, kindly provided by Dr. Valentina Monica, Department of Oncology, University of Torino, AOU San Luigi Gonzaga, were cultured in Gibco BenchStable RPMI 1640 GlutaMAX medium (Thermo Fisher) supplemented with 10% Fetal Bovine Serum (FBS) and 1% penicillin/streptomycin (PS) (all from Sigma–Aldrich). Cells were cultured in a humidified incubator at 37 °C with 5% CO_2_, and they were periodically checked for mycoplasma contamination.

Conditioned medium (CM) experiments were conducted by pouring 150 μL of the sterilized liquid resin into a 48-well plate (TC untreated, Greiner Bio-One) and cured using the DLP’s LED for 10 min, followed by a postcuring step of 5 min (UV light 365 nm). Hydrogels were then washed with 800 μL of sterile PBS for 24h at 37 °C. Then, after PBS removal, 800 μL of the required cell-culture medium was conditioned for 96 h at 37 °C. CM was collected and stored at 4 °C until needed.

For viability assessment, 4 × 10^3^ A549 were seeded onto a 96-well plate (TC treated, Greiner Bio-One) in a conditioned medium and in a complete medium as the negative control (200 μL for each well). Cells were then incubated, and after 24 and 96 h viability was determined by using 200 μL of 0.5 mg/mL of 3-(4,5-dimethylthiazol-2-il)-2,5-diphenyltetrazolium bromide (MTT, Sigma-Aldrich, M2128) solution dissolved in a complete medium. After 2 h of incubation at 37 °C, the MTT solution was removed, and formazan salts were dissolved in 200 μL of DMSO. Afterward, optical density (OD) at a wavelength of 570 nm (reference at 650 nm) was measured by a Synergy HTX Multi-Mode Microplate Reader (BioTek, Winooski, VM, USA). Each test was carried out at least three times.

#### 3D Cell Culture

A549 cells were suspended in the sterile formulation at a final concentration of 1.5 × 10^6^ cells/mL. Cell-laden cylindrical constructs were fabricated using a digital light processing (DLP) 3D bioprinter (BIONOVA, CelLink, BICO Company) operating in continuous printing mode with a 405 nm LED set to 100% intensity and a printing speed of 0.012 mm/s. After printing, the excess uncured formulation was removed, and the samples underwent one step of washing in PBS. Culture medium was then added, and the printed scaffolds were incubated under standard cell culture conditions.

Resazurin assay (Sigma-Aldrich) was performed on cell loaded bio printed scaffold after 1 or 4 days of culture. Briefly, 100 μL of resazurin (0.1 mg/mL in complete medium) was added to each well and incubated at 37 °C for 1.5 h. The fluorescence signal (exc/em: 530/590) was always detected by the SynergyTM HTX Multi-Mode Microplate Reader.

Statistical analysis was performed using a one-tailed *t* test to assess the significance of differences between groups. p-values were considered statistically significant as follows: *<0.05; **<0.01; ***<0.001.

The viability of cells embedded in printed samples was assessed by a Live/Dead staining kit (Sigma–Aldrich). Specifically, reagents were dissolved according to the manufacturer’s instructions, and samples were incubated at 37 °C in the staining solution for 40 min. After a washing step in DPBS, the constructs were visualized with a microscope (Eclipse Ti2 Nikon, Tokyo, Japan) equipped with a Crest X-Light spinning disk confocal microscope and a Lumencor SPECTRA X light engine.

To evaluate cell morphology, printed samples were washed with DPBS and permeabilized with a solution of TRITON X-100 0,1% v/v for 15 min. Cytoskeletal and nuclei were stained for 1 h with Phalloidin-FITC (Thermo Fisher) and DAPI (Sigma-Aldrich) at the concentrations of 0.25 and 2.5 μM, respectively. After three final washing steps, the constructs were visualized with the same microscope at 20x magnification.

### Swelling Behavior

The study for swelling and stability of 3D-printed samples was carried out in different solutions to verify the amount of liquid the hydrogel is able to retain and to ensure its applicability in biomedical applications. The sample of known weight was dipped in 1 mL of solutions such as phosphate buffer, deionized water, pH solutions of acidic and basic form (Tris-HCl buffers with pH 5, pH 7.4, and pH 9, respectively), solutions with different glucose concentration (0.5, 5, and 50 mM) and incubated at 37 °C; its weight change was observed for 14 days. The % weight change was evaluated using the formula given in eq 2:

$$\%W_{s}=\frac{W_{f}-W_{i}}{W_{i}}\times 100$$ 2

where *W*_f_ is the final weight and *W*_i_ is the initial weight. For each solution, three parallel readings were taken, and an average value was considered as the final % weight change.

## Results and Discussion

The initial aspects to consider for VAT-based 3D printing are the processability of the liquid formulations, the photopolymerization kinetics, and the properties of the resulting hydrogels. To complete this preliminary characterization, a series of rheological analyses was performed.

DLP-based printing requires resins with high flowability; thus, the viscosity of the formulations was assessed to verify their compatibility with the printing process (Figure 1c). As shown in Table S1, viscosity decreases with increasing temperatures, although minimal changes were observed between 25 and 35 °C. The temperature ramp test (Figure S2a) demonstrated a gradual increase in the storage (G′) and loss (G″) moduli above 30 °C, reaching a plateau around 40 °C. These results indicate that adequate fluidity can be achieved for 3D printing at RT without the need for additional thermal control.

Preliminary amplitude tests were conducted at 1 Hz on the liquid formulations (Figure 1b) to determine the appropriate strain value to ensure measurements within the linear viscoelastic region (LVE). Based on these results, a strain amplitude of 50% was chosen for subsequent photorheology experiments. In addition, frequency sweeps performed on the liquid formulation (Figure 1c) confirmed the characteristic viscoelastic behavior of the precursor solution before gelation.

Thus, measurements were performed at 1 Hz and 50% strain, and the results are shown in Figure 1d: under UV irradiation, a latency time of 7–10 s before the beginning of polymerization reaction was observed, followed by a rapid increase in G′ and G″, with the gel point (G′ = G″) occurring within 15–20 s. G′ stabilizes at about 120 kPa after 2.5 min of exposure, a significantly higher value than typically reported for similar systems (G′∼10^4^ Pa).^3,99,100^ The corresponding cross-link density (ν_e_), calculated with eq 1, was estimated to be ∼10^25^ m^–3^. The results highlight the efficiency of the photopolymerization process and, additionally, give preliminary insights into the mechanical properties of the obtained hydrogels, suggesting their high potential for application in 3D printing. The key photorheological parameters are summarized in Table S2.

Finally, the mechanical properties of the hydrogels were further studied through frequency and amplitude sweep tests. Frequency sweeps (Figure 1e) revealed a frequency-dependent viscoelastic response, which is frequently noted in networks with dynamic covalent bonds. At high frequencies, G′ exceeds G″, indicating a predominantly elastic behavior typical of chemically cross-linked systems. In contrast, at low frequencies, G″ exceeds G″, consistent with the viscoelastic relaxation of a transient network governed by dynamic borate-diol interactions. Amplitude sweeps on gelled samples (Figure 1f) showed constant G’ and G’’ up to a critical strain, beyond which G′ decreases, indicating an initial network failure. This critical strain exhibited a marked temperature dependence, being higher at lower temperatures (8% at 5 °C, 1.5% at 25 °C, 0.5% at 37 °C; Figure S 2d). At lower temperatures, the loss modulus G″ exhibits a pronounced peak upon network disruption, attributable to the formation and growth of microfractures within the network, leading eventually to macroscopic failure and fluid-like behavior (G″ > G′).

Once the rheological characterization of the material was completed, the formation of borate ester bonds in hydrogels was evaluated by using both solid-state NMR and FT-IR spectroscopy.

The SS-NMR study conclusions presented here are based on the detailed analyses of both the precursors and a set of suitably prepared samples, which are reported in the Supporting Information (Figure S3). Figure 2a shows the ^13^C NMR spectrum of the hydrogel, whose simplified schematic structure is reported in Figure 2b to highlight the main functional groups discussed below. The absence of signal due to vinyl groups and the presence of methylene resonances (50–40 ppm) suggest a successful copolymerization between HEMA and PEGDA.^101–103^ This is further supported by the chemical shift and the line shape of the C=O resonance (180–170 ppm). The presence of the sharp resonance at 28 ppm can be attributed to the S bridges between DTT and the copolymer. Moreover, the methylene resonance of DTT appears downfield shifted and split into two peaks at 35.7 and 37.3 ppm, indicating formation of borax/DTT complexes and reaction with the copolymer.^104,105^ The downfield shifted DTT methine resonance (76 ppm) further indicates the occurrence of bonding with borax.

**Figure 2:**
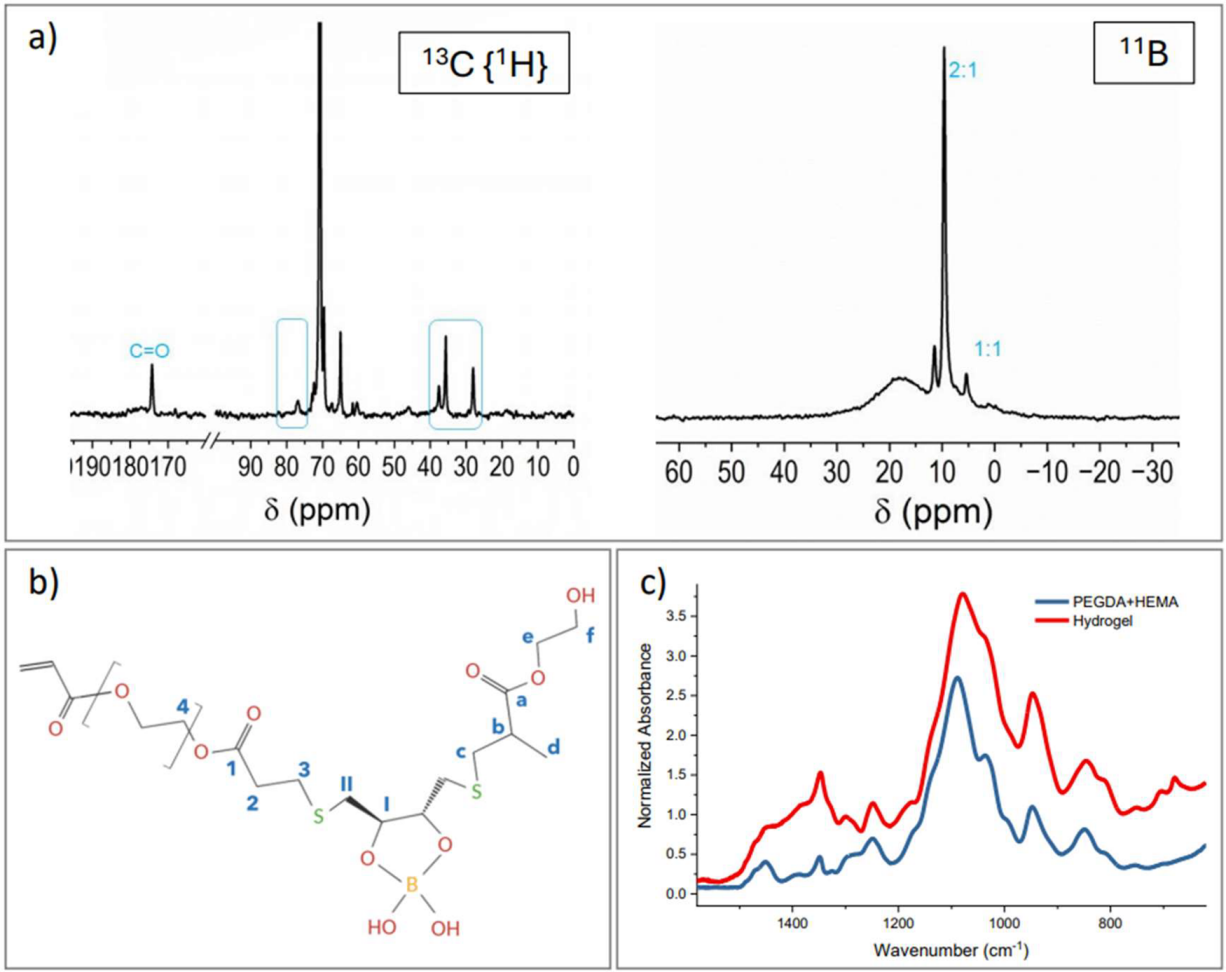
(a) Hydrogel’s 13C {1H} decoupled MAS NMR (on the left) and 11B MAS NMR (on the right): signals at 35.7, 37.3, and 76 ppm are consistent with boron coordination to DTT, supporting the formation of borate-based cross-links; resonances at 9.6 and 5.1 ppm, confirm the presence of borate-DTT complexes; (b) simplified schematic structure of the hydrogel with C labeling and (c) FTIR spectra of the hydrogel formulation (red curve) compared to polymerized PEGDA + HEMA (blue curve): the presence of borate ester bonds is evidenced by the broad peak at 1400 cm^–1^; in addition, the appearance of two peaks at 705 and 680 cm^–1^, attributable to B–O and C–S bonding, respectively, can be noted. Spectra were normalized with respect to the 1720 cm^–1^ peak, corresponding to ester groups (−COOR).

The ^11^B NMR spectrum (Figure 2a) further confirms the reaction of DTT with borax, with the formation of 2:1 and 1:1 complexes, as indicated by the sharp peaks at 9.6 and 5.1 ppm, respectively. The broad resonance at 18 ppm is attributed to B­(OH)_3_/[B­(OH)_4_]^−^ species in equilibrium due to the overlapping of chemical shifts and the dynamic exchange processes between them on the NMR time scale.^3,104–106^ Finally, the sharp resonance at 11.2 ppm may correspond to adducts,^107^ possibly arising from reaction with the many hydroxyls of the radical scavenger.^105,107^

FTIR spectroscopy confirmed these results. In the DTT-Borax complex (Figure S4a), borate ester formation is evidenced by a strong absorption band in the region between 1300 and 1400 cm^–1^.^108^ Other bands attributed to borate ester bonds were also noted: in particular, the regions between 1220 and 1260, 1000–1060, and 550–700 cm^–1^ were assigned to C–O stretches, B–O stretches, and out-of-plane vibrations, respectively.^109^

The presence of borate–ester bonds is also confirmed by the final hydrogel’s FTIR spectrum, compared to the one of the copolymer PEGDA + HEMA (Figure 2c): in particular, there is an increase in signal in the 1350–1450 cm^–1^ band, which can be attributed to the O–B–O stretching of the borate-ester bond.^108^ In addition, two peaks appear at 705 and 680 cm^–1^, which can be attributed to B–O and C–S bonding, respectively.^110,111^

Once the presence of dynamic covalent bonds in the hydrogel structure was confirmed, 3D printing by DLP was carried out. The printing parameters were optimized in order to achieve a compromise between the resolution and printing speed. Different structures were 3D-printed, ranging from simple to more complex shapes (Figure 3a). The addition of a radical scavenger improved printing resolution by limiting polymerization to the irradiated regions, thus preventing undesired propagation in nonexposed areas.^112^ Printing parameters were optimized and are detailed in the Materials and Methods section. As part of the formulation characterization, two 3D-printed benchmarks were produced to assess the achievable printing resolution (Figure 3b,c). The first structure included a series of pillars with decreasing dimensions of 2, 1, 0.5, 0.25, and 0.1 mm. All pillars were successfully printed; however, the 0.1 mm one appeared to be poorly defined. Based on these observations, the minimum achievable resolution in the xy plane can be estimated to be 250 μm. The second structure consisted of a series of pillars printed at specific distances (5, 3, 2, and 1 mm), and showed a *z* resolution of approximately 1 mm.

**Figure 3:**
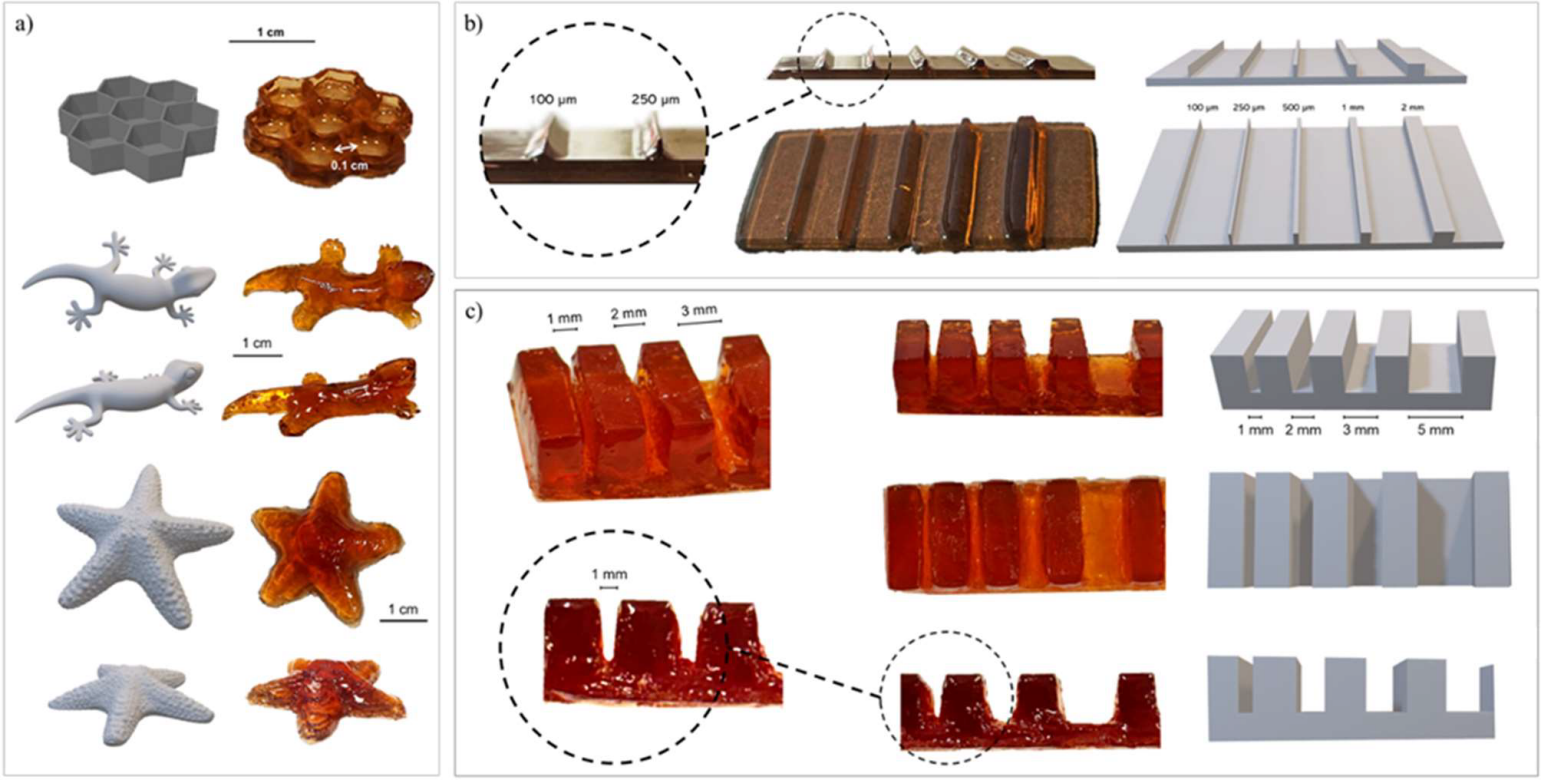
(a) Comparison between original CAD models and corresponding 3D printed structures, demonstrating high printing fidelity. (b) CAD and 3D printed benchmark structure, consisting of pillars with varying thickness (2, 1, 0.5, 0.25, and 0.1 mm); all pillars are fully printed, however, the 0.1 mm pillar appears poorly defined, as highlighted in the magnified view, indicating an effective lateral resolution (*xy*) of approximately 250 μm; (c) CAD and 3D-printed benchmark structure, consisting of arrays of lines printed at specific distances (5, 3, 1, and 1 mm), showing a z resolution of approximately 1 mm.

Considering the presence of reversible dynamic-covalent bonds within the hydrogels due to the borax/diol complex, we studied the self-healing characteristics of the hydrogels. Both qualitative and quantitative tests were conducted. As a qualitative assessment of self-healing, cut-and-healing tests were conducted on 3D-printed rectangular specimens, which were cut into two pieces and rejoined to allow repair at room temperature. Repair capability was evaluated by assessing the visibility of the cutting interface, the ability of the specimen to support its own weight, and the ability to be bent and stretched. To determine the time required for healing, preliminary tests were performed by staining one-half of the specimens with Brilliant Green dye and evaluating, in addition to the other parameters, the diffusion of the dye from one side to the other. Samples were observed at different times: At 24 h, no healing capacity was noted, whereas already at 48 h, the specimen could support its own weight. After 72 h, not only does the specimen sustain its own weight, but better diffusion of the dye and the ability to be pulled by breaking at a point other than the original fracture line are noted (Figure 4a). Therefore, 72 h was chosen as the minimum amount of time to allow adequate healing of the hydrogels. Similar tests have been repeated on 3D printed samples of even more complicated geometries, confirming the maintenance of the repair capability (Figure 4b). Comparisons between samples with and without borax were carried out, which showed that the self-repair ability was attributable to dynamic borate–ester bonds, as expected. More information can be found in the Supporting Information (Figure S5).

**Figure 4:**
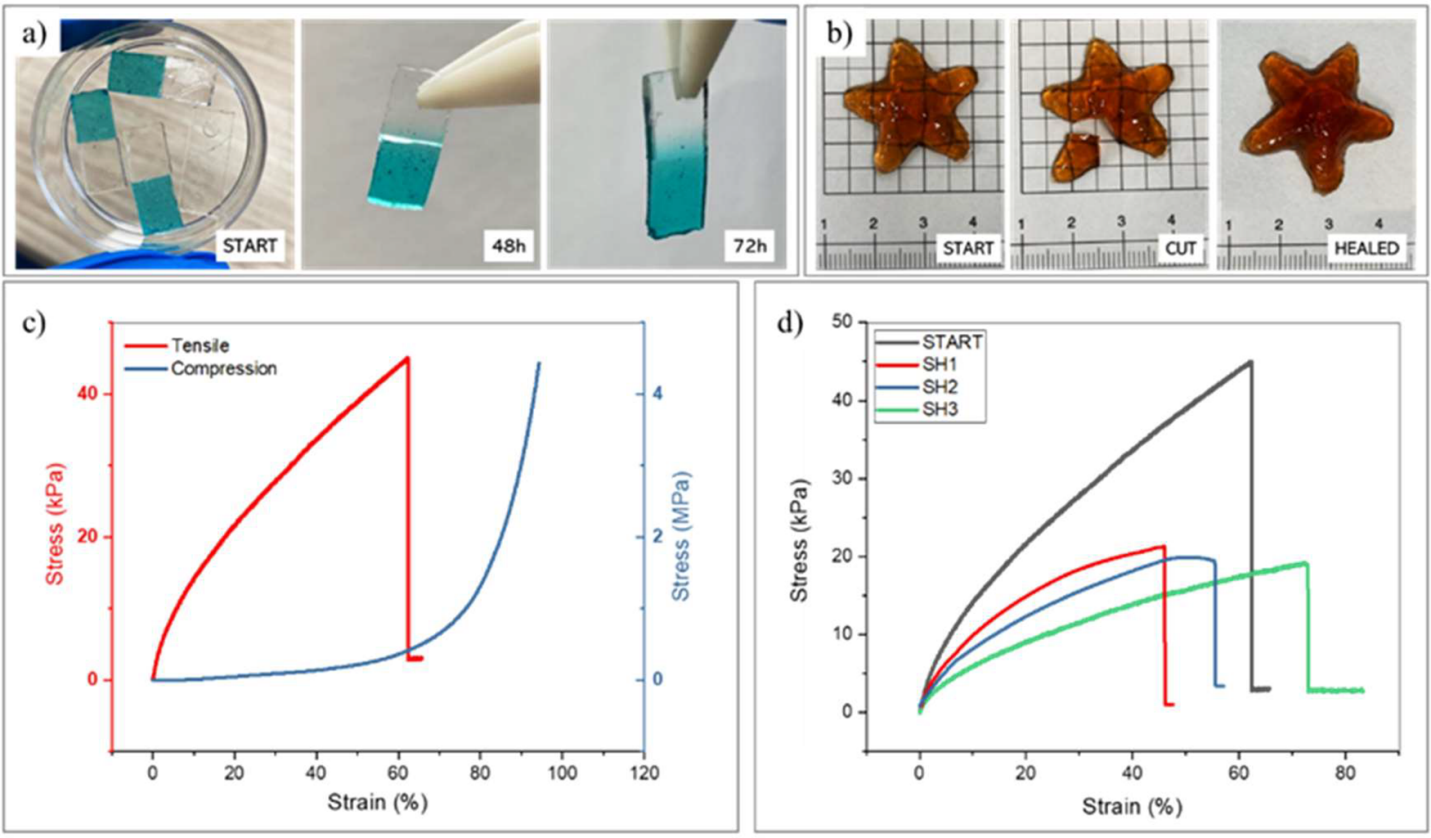
Self-healing behavior and mechanical performance of the printed material. (a) Healing test of cut samples, where half was colored with a dye to visualize the interfacial diffusion. After 48 h, partial diffusion and mechanical integrity are observed, while after 72 h the interface is more uniform and capable of supporting the sample’s weight, indicating improved healing. (b) 3D-printed star-shaped sample undergoing a healing test. The initial cut is no longer visible after 72 h, demonstrating effective healing even in complex geometries. (c) Representative stress–strain curves from tensile (red, left *y*-axis) and compression (blue, right *y*-axis) tests. In tension, the material shows an elastic modulus of ∼260 kPa and elongation up to ∼60%; in compression, the elastic modulus is ∼60 kPa with a strain up to ∼90%. (d) Stress–strain curves after repeated self-healing cycles. Compared to the fresh sample (black curve), mechanical performance progressively decreases with each cycle, but the material retains the ability to deform up to ∼80% strain even after three healing events (green curve).

SEM analysis of self-healed samples (Figure 5) allows visualization of the restoration of the materials after damage (complete cut). To better evidence it, a slight misalignment was present in the self-healed one, which helps to visualize the healed volume. Notably, no cracks are present, and the surface is homogeneous and smooth as in the control specimens, confirming the efficacy of the proposed strategy.

**Figure 5:**
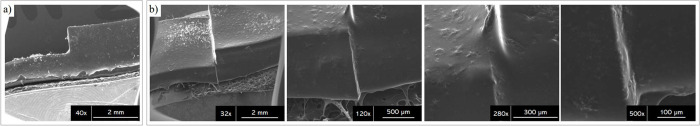
SEM images showing the self-healing in DLP-printed hydrogels. (a) Control specimen, not subjected to damage; (b) specimen after going through the self-healing process with increasing magnifications. The images show restored continuity at the damaged interface, indicating successful healing of the polymer structure.

Quantitative analysis was conducted not only to evaluate self-healing capability but also to mechanically characterize the hydrogels; tensile and compressive mechanical tests were conducted. Specifically, the value of elastic modulus and elongation at break, through several cycles of breaking and healing, was assessed. Representative results of tensile and compression tests are shown in Figure 4c; the mean value of the mechanical features of the hydrogel is reported in Table 2.

**Table 2:** Mechanical Parameters in Tension and Compression Modes^a^

	**compression**		
	elastic modulus (kPa)	strength (kPa)	elongation at break (%)
start	56 ± 21	3720 ± 440	92.2 ± 2.7

	tensile		
	elastic modulus (kPa)	strength (kPa)	elongation at break (%)
start	259 ± 46	39 ± 8	62 ± 21
after 1st SH	182 ± 38	20 ± 8	45 ± 26
after 2nd SH	147 ± 34	14 ± 5	44 ± 17
after 3rd SH	113 ± 11	15 ± 4	79 ± 4

a In the case of tensile mode, mechanical parameters after self-healing are also shown.

Under tensile stress, hydrogels show good mechanical properties with an elastic modulus of around 260 kPa, an elongation at break of 62%, and a maximum strength of ∼40 kPa. Tensile tests were repeated after 72 h, also on the specimens brought to failure, to evaluate the mechanical properties after self-healing. After three consecutive cycles of damage and self-repair, a progressive reduction in stiffness and strength was observed: the modulus decreases to 44% of the initial value, while the ultimate tensile strength drops by about 60%. Despite this, the material retains a high deformation capacity; in fact, in the third cycle, the elongation at the break is 26% greater than the original value, suggesting a transition to more ductile behavior. This phenomenon can be explained by an effective microstructural reorganization that occurs during the self-healing process, sustained by the reformation of reversible bonds capable of restoring the continuity of the polymer matrix even in the presence of damage.

To distinguish the effects of mechanical damage from those of aging, specimens not subjected to mechanical damage but left to age for equivalent periods were analyzed. The results show that aging also results in a decrease in stiffness and strength (up to −76% in elastic modulus and −60% in ultimate tensile strength) but at the same time causes a systematic increase in ductility. Direct comparison between self-repaired and aged specimens shows that, initially, the healing process promotes a partial recovery in stiffness but at the expense of a greater loss in strength and elongation compared with simple aging. However, over longer time scales, the mechanical properties of both sets of specimens tend to converge, suggesting that long-term degradation is primarily governed by aging mechanisms. These findings show that the overall mechanical degradation is linked both to the natural effects of aging and to some microstructural modifications induced by the self-healing process, which has a tendency to form a stiffer but weaker polymer matrix. A specific comparison of self-healing and aging data can be found in the SI (Figure S6).

To better mimic biomedically relevant conditions, the hydrogels were swelled in water for up to 14 days and then characterized. The material maintains very good dimensional and chemical stability: after 14 days of immersion, despite the considerable reduction of mechanical properties, samples did not dissolve or break; this was expected considering that the used materials are synthetic polymers, which usually undergo cleavage of bonds in very basic conditions.^113^ In addition, application of the self-healing process to samples swelled for 1 day revealed remarkable functional recovery: although the strength remains modest (ca. 45% of the initial value), elongation at break basically returns to its initial values (63%), indicating that the dynamics of bond reformation in the polymer network work also in an aqueous environment.

In compression tests, the material shows a lower elastic modulus (about 56 kPa) but is extremely ductile, with deformation at break higher than 90% and compression strengths on the order of MPa. Even for this type of testing, the effects of aging and swelling on compressive mechanical properties were studied.

Due to aging, the material becomes stiffer, with an increase in E up to about 150 kPa after 14 days of aging. At the same time, there is a reduction, although not significant, in the deformation at the break. This behavior can be consistent with a slight change in the polymer network of the polymer, with a possible variation of chemical/physical cross-linking point, maybe related to the slight evaporation of the solvent. In any case, the material resulted in stiffer and more compression-resistant, even if with a slight reduction in deformability.

In contrast, swelling in water results in a reduction in strength and deformability, as we expected. After 1 and 7 days of immersion, the elastic modulus is reduced by about 45% and elongation by 30%, while the mechanical strength drops dramatically to about 150 kPa. After 14 days, there is a partial increase in elastic modulus, probably due to aging of the specimen.

Together, the results demonstrate that the material is capable of withstanding several damage cycles and resisting swelling over long intervals with sufficient mechanical response for practical applications. The synergy between effective self-repair and swelling resistance demonstrates the promise of the material for applications in dynamic and humid environments, such as biological ones.

Afterward, to evaluate the potential of the developed hydrogels for biomedical applications, a biocompatibility analysis was carried out. Initial analysis focused on the evaluation of the potential leaching of cytotoxic substances derived from incomplete polymerization, so cell viability was assessed after exposure to cell culture medium, previously conditioned by polymerized hydrogels. Specifically, the response of human lung adenocarcinoma cells (A549) was examined: viability assays revealed a slight increase in cell growth between 24 and 96 h, even if this increase was less pronounced compared to the control group. This result is likely not due to toxicity but rather to the sequestration of essential nutrients from the media by the hydrogels during conditioning. Detailed results are shown in the SI (Figure S7).

Hence, to better characterize the cytocompatibility of the designed hydrogels, direct encapsulation of A549 cells in the formulation was performed. Specifically, the cells were dispersed in liquid resin before the printing process. Thus, with this approach, viability after irradiation and inclusion was monitored without affecting the availability of the nutrient, and by providing external stimuli typically found under physiological conditions. Notably, the encapsulated cells exhibited a good proliferation rate and a significant increase from 24 to 96 h postprinting (Figure 6a). These findings demonstrate that neither the DLP-printing process nor the photo-cross-linking compromises cell viability, confirming the biocompatibility of the design hydrogel. Further characterization by nuclei and cytoskeletal staining unveiled that the cells were homogeneously dispersed into the hydrogel, maintaining the expected morphology in a 3D environment (Figure 6b). Finally, the maintenance of cell viability after 96 h from the procedure (Figure 6c) demonstrated the absence of delayed toxicity. Collectively, these findings showed how the printing process and hydrogel nature did not interfere with cell behavior, making this platform capable of supporting the fabrication of complex and cell-loaded scaffolds, opening up the possibility for a plethora of biomedical uses.

**Figure 6:**
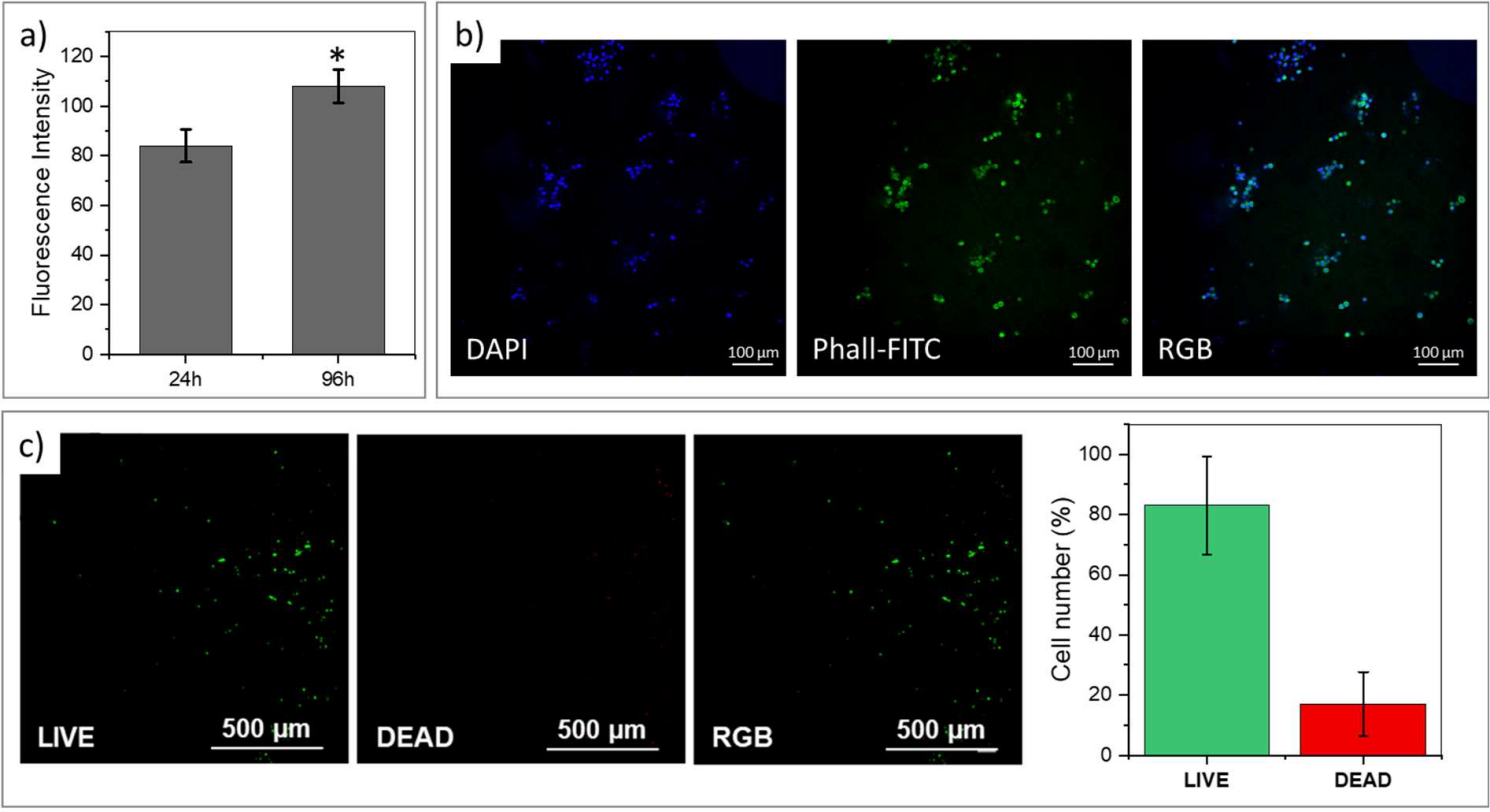
(a) Resazurin assay on 3D printed hydrogels containing embedded cells after 24 and 96 h (*p*-value *<0.05; **<0.01; ***<0.001); fluorescence intensity measured at 24 and 96 h suggests cell proliferation over time. Control corresponds to the fluorescence of cell-free hydrogel incubated with resazurin. (b) Representative picture of nuclei (DAPI, Blue) and cytoskeletal (Phall-FITC, green) staining performed after 96 h of culture. Scale bar: 100 μm. (c) Live and dead assay performed on cell-laden 3D printed hydrogels 96 h after printing, that demonstrates cell viability.

Having demonstrated the suitability of the hydrogels in the biological field, their behavior upon swelling was further investigated in different solutions, varying pH, and glucose concentrations. This study was conducted not only to mimic different physiological environments but also because the DTT–borax cross-linking is known to be sensitive to changes in both pH and glucose levels. The aim was to assess the stability of the hydrogels under these conditions in view of potential biomedical use. The results of swelling tests are shown in Figure S8 and Table S5.

Similar swelling in PBS and in a pH 7.4 solution was observed within the first 3 h, with values close to 50% of swelling. For longer observation times (14 days), PBS and pH 7.4 continue to have similar behavior: the hydrogel reaches a plateau at ≈90% swelling after 4 days, and its structural integrity remains stable during the whole observation period, indicating that no degradation occurs. Under basic conditions, a rapid initial swelling of the hydrogel was observed, reaching approximately 140% within the first 24 h, with an estimated initial rate of ∼19%/h. This was followed, however, by a marked degradation of the hydrogel network with a mass loss of ≈57% at day 4. Such behavior can be attributed to the instability of borate–ester linkages in strongly alkaline environments, where hydrolysis of these dynamic covalent bonds is accelerated, ultimately leading to network breakdown.^114^ In contrast, under acidic conditions, the hydrogel exhibited minimal swelling, around 10–20% in the first 24 h, and degradation was observed later compared to basic conditions, with the hydrogel returning to its initial weight after day 7 and losing ≈60% after day 14. The reduced swelling at low pH may be due to the protonation of borate species, which diminishes their ability to interact with diol-containing cross-linkers such as DTT, thereby limiting hydrogel hydration and network expansion.^114^ Similar swelling in PBS and in a pH 7.4 solution was observed within the first 3 h, with values close to 50% of swelling. For longer observation times (14 days), PBS and pH 7.4 continue to have similar behavior: the hydrogel reaches a plateau at ≈90% swelling after 4 days, and its structural integrity remains stable during the whole observation period, indicating that no degradation occurs. Under basic conditions, a rapid initial swelling of the hydrogel was observed, reaching approximately 140% within the first 24 h, with an estimated initial rate of ∼19%/h. This was followed, however, by a marked degradation of the hydrogel network, with a mass loss of ≈57% at day 4. Such behavior can be attributed to the instability of borate–ester linkages in strongly alkaline environments, where hydrolysis of these dynamic covalent bonds is accelerated, ultimately leading to network breakdown.^114^ In contrast, under acidic conditions, the hydrogel exhibited minimal swelling, around 10–20% in the first 24 h, and degradation was observed later compared to basic conditions, with the hydrogel returning to its initial weight after day 7 and losing ≈60% after day 14. The reduced swelling at low pH may be due to the protonation of borate species, which diminishes their ability to interact with diol-containing cross-linkers such as DTT, thereby limiting hydrogel hydration and network expansion.^114^

With respect to glucose concentration, its influence on the swelling behavior of borate-cross-linked hydrogels has been previously reported.^3,33^ Depending on polymer composition and environmental conditions, glucose can induce either swelling or deswelling.^3,115^ In the present system, over the early short observation period (first 3 h), there were no significant differences, with all the samples reaching ≈90% swelling, while a different behavior was observed for longer time scales. In a high concentration of glucose environment (50 mM), the hydrogel reaches ≈90% swelling within the first 3 days and then undergoes steady deswelling, coming down to ≈24% after 14 days. At the intermediate concentration (5 mM), swelling is increased to ≈120% within 3 days and then remains stable, while at a lower concentration (0.5 mM), the hydrogel shows maximum swelling, reaching ≈175% after 1 week and maintaining the same value up to 14 days. This phenomenon is consistent with findings by Alexeev et al.,^116^ who reported that glucose can bind simultaneously to two borate units within the hydrogel network, thereby increasing cross-linking density and reducing swelling. Similar deswelling effects upon glucose exposure have also been observed in phenylboronic acid (PBA)-functionalized hydrogels.^117–119^

## Conclusions

In this study, a self-healing hydrogel system based on reversible borate–ester bonds between borax and diols was developed and characterized. The dynamic nature of these interactions endowed the material with self-healing capabilities, which were maintained for multiple cycles of damage healing, even with a progressive decrease in mechanical performance. However, the elastic modulus remained in the tens of kilopascals range, making the material suitable for soft biomedical applications. The hydrogel was successfully processed by DLP (Digital Light Processing) 3D printing, enabling the fabrication of geometrically complex structures with good shape fidelity and an estimated lateral resolution of about 250 μm. The formation of borate–diol ester bonds within the hydrogel network was confirmed by FTIR and solid-state NMR, while the dynamic nature of the cross-linking was evidenced by self-healing experiments.

Preliminary biological analysis demonstrated the overall suitability of the designed hydrogel in supporting cell viability and growth, suggesting future application in the biomedical field. In addition, the swelling behavior of the hydrogels was evaluated under various pH and glucose conditions, mimicking physiologically relevant environments. These studies confirmed that the material remains stable in neutral conditions but undergoes controlled degradation in acidic or basic environments, a feature that could be advantageous for drug delivery or tissue engineering applications.

Together, these results support the potential of this boric acid-based hydrogel system for biomedical use, particularly in cases where dynamic mechanical properties, moldability, and biocompatibility are required. In addition, the borate-ester mechanism here described is a versatile approach for introducing self-healing functionality into various systems due to its simplicity, aqueous compatibility, and absence of cytotoxic reagents. This chemistry can potentially be used in other matrices or combined with other functionalities such as bioactivity, degradability, or stimuli-responsiveness, opening the door to future uses of self-healing biomaterials, as well as new biofabrication approaches.

Future work will focus on the exploitation of the self-healing potential of cell-loaded hydrogels to better define the potentialities of this novel approach in studying the dynamics of a complex microenvironment. Indeed, this method would clear the way for the development of functional, cell-loaded architectures suitable for regenerative medicine, *in vitro* tissue modeling, or bioactive implantable devices. Further formulation optimization will also be pursued to balance printability, mechanical integrity, and biological functionality in complex biological environments.

## Supplementary Material



## Notes

Schemes of the chemicals employed and UV–vis spectrum of LAP; formulation design, NMR and FT-IR analysis comments; additional rheology studies; compositions of the studied formulations; NMR and FT-IR additional graphs; images of the effect of borax in the formulations; additional mechanical tests; additional MTT results; and swelling behavior data (PDF)
