# Supplementary material for: Biocompatible Self-Healing Hydrogel for VAT 3D Printing
Source: ACS Mater Au. 2026 Feb 13;6(3):539–52. doi: 10.1021/acsmaterialsau.5c00194 (PMC13177411; doi:10.1021/acsmaterialsau.5c00194)
Supplement: Supplementary file 1 [file mg5c00194_si_001.pdf]

SUPPORTING INFORMATION

Biocompatible self-healing hydrogel for VAT 3D printing

Maria D'aloia[1,2,3], Désirée Baruffaldi[1,3], Sandra Dirè[4], Emanuela Callone[4], Angelo Angelini[5], Candido Fabrizio Pirri[1,3,6], Ignazio Roppolo[1,3,6*], Francesca Frascella[1,3]

[1] Department of Applied Science and Technology, Politecnico di Torino, Corso Duca degli Abruzzi 24, Turin 10129, Italy

[2] Department of Chemistry, Biology and Biotechnology, University of Perugia, Via Elce di Sotto 8, Perugia 06123, Italy

[3] PoliTOBioMEDLab, Politecnico di Torino, Corso Duca degli Abruzzi 24, Turin 10129, Italy

[4] "Klaus Müller" Magnetic Resonance Lab, Department of Industrial Engineering, University of Trento, Via Sommarive 9, Trento 38123, Italy

[5] Italian National Institute of Metrological Research (INRiM), Strada delle Cacce, 91, Turin 10135, Italy

[6] Center for Sustainable Future Technologies, Italian Institute of Technology, Via Livorno 60, Turin 10144, Italy

Formulation design:

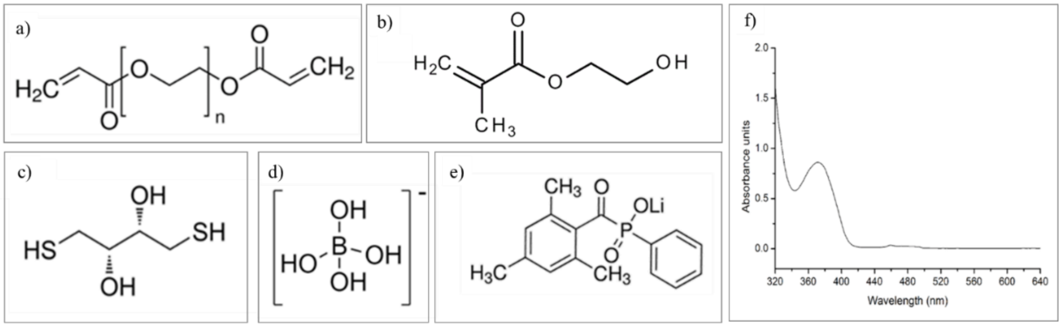

Figure S 1. Chemical structures of (**a**) PEGDA, (**b**) HEMA, (**c**) DTT, (**d**) borax, (**e**) LAP and (**f**) absorbance spectrum of LAP.

The hydrogel formulation was developed starting from the work of He et al.[1], who reported a PEGDA-based system incorporating dithiothreitol (DTT) and borax, capable of spontaneous gelation and exhibiting self-healing properties due to the formation of dynamic borate–ester bonds. In the present study, the original formulation was modified by introducing 2-hydroxyethyl methacrylate (HEMA), with the purpose of inhibiting spontaneous crosslinking, as a reactive diluent to decrease the viscosity of the formulation[2], and to provide additional acrylate groups for controlled photopolymerization, together with PEGDA. Moreover, the hydroxyl groups from HEMA contribute to the formation of physical crosslinks via interaction with borax. The molar ratios between DTT and acrylates, as well as between DTT and borax, were systematically optimized to achieve a balance among printability, printing resolution, mechanical performance, and self-healing capacity of the resulting hydrogel.

PEGDA gives structural integrity to the matrix, building cross-linked networks thanks to acrylate groups that enable free-radical polymerization[3]. PEGDA is known for its hydrophilicity, curing speed and dimensional stability[3,4], and several studies demonstrated the low cytotoxicity of polymerized PEGDA, although the base monomers are instead extremely toxic[3–6]. PEGDA-based materials have enabled the development of numerous devices for use in various biomedical applications[5,6], and PEGDA-based hydrogels in particular have been extensively studied[5,7–9]. HEMA is a biocompatible, photopolymerizable monomer with tunable mechanical properties[10]. Poly-HEMA-based hydrogels have often been used as devices for biomedical applications[11–14].

To achieve the borate ester chemistry, responsible for the self-healing ability, borax and DTT are added. In its ionic form, borax presents an anion called tetrahydroxyborate which is a boron atom bonded to four hydroxyl groups. In the presented work, DTT serves as a copolymer, binding to acrylate chains through its two thiol groups, while its exposed hydroxyl groups form borate ester bonds with tetrahydroxyborate's hydroxyl groups via transesterification reaction. This reaction can occur twice on both sides of the ion, involving two molecules of DTT present in two adjacent polymer chains, leading to the formation of inter-chain bridges capable to associate and disassociate reversibly. PBS (pH between 7-7.4) was chosen as the liquid phase to help maintaining a constant pH.

After adding a DTT-borax solution to a homogeneous aqueous solution containing acrylates, the formulation is printed through DLP and a hydrogel was obtained. The proportions of borax, DTT and acrylates were studied in order to achieve the right compromise between mechanical, self-healing and printability properties. A photoinitiator is needed to achieve the photopolymerization process, and we also added a radical scavenger to improve the printing resolution. In a radical photoinduced process, radical scavengers may react both with initiating species and propagating species, inducing termination. If on the one hand RSs reduce the speed of the 3D printing process by decreasing the rate of polymerization, on the other hand they can interact with growing macro-molecules, that are generated in the irradiated voxels and then propagate out of those, stopping the polymerization. A balanced introduction of RSs can thus slightly affect the polymerization rate but efficiently end propagation, confining polymerization in the irradiated area and increasing resolution.

To confirm that the observed self-healing behavior is specifically mediated by the borax–DTT dynamic interaction, control samples were prepared using the same protocol but without borax. These samples were cut in half and the surfaces were brought back into contact; samples were then maintained under the same conditions as the experimental group. The evolution of healing was qualitatively assessed at 48, and 72 hours. In contrast to the borax-containing hydrogels, which exhibited the ability to support their own weight after 48 hours and regained tensile integrity by 72 hours, the control samples without borax showed no signs of self-repair. The cut interface remained clearly visible, the diffusion of the dye is minimal and the two halves failed to adhere or recover any mechanical continuity over time. These results confirm that the self-healing capacity of the hydrogel relies on the reversible borate–diol interactions enabled by the presence of borax in the network (Figure S5).

<u>Structural analysis:</u>

To investigate the structural characteristics of the hydrogel and to confirm the formation of borate–ester bonds, a spectroscopic analysis was conducted through Fourier-transform infrared (FTIR) and solid-state nuclear magnetic resonance (ssNMR) spectroscopy. In order to correctly assign the spectral resonances and verify the expected structural modifications, a series of reference samples were prepared and analysed. These included individual components (DTT, borax, and their complex) as well as progressively built hydrogel formulations composed of PEGDA, HEMA, DTT, and borax in phosphate-buffered saline (PBS). This stepwise strategy facilitated the observation of spectral changes correlated with the formation of covalent and non-covalent interactions within the hydrogel network. The details of the analysed samples, including sample labelling and compositions, are provided in Table S3.

<u>SS-NMR</u>

Figure S 3 shows the spectrum of the final hydrogel formulation, here labelled as PHDBEV, along with all the other samples listed in Table S3.

Two broad resonances in the spectrum of the DB sample, due to ethylene (35-25 ppm) and methine (80-70 ppm) groups with asymmetric lineshapes, suggest the presence of both free DTT (**I, II**) and borax-complexed DTT (**I', II'**) in different stoichiometries[15]. Approximately two-thirds of DTT hydroxyl groups seem to be reacted with boron. The DB reference sample exhibits three resonances: a broad one at $\delta_{iso} \approx 18$ ppm, with anisotropy due to a non-zero quadrupole coupling constant and asymmetry parameter, and two Lorentzian peaks at 9.2 and 0.5 ppm. They are assigned to $B(OH)_3$ species, a 2:1 diol/monoborate complex, and $B(OH)_4$[illegible] species, respectively[15].

Both HEMA and PEGDA appear to be fully polymerized, as indicated by the absence of double bond resonances in the 135–128 ppm region and the presence of methylene signals **b'**, **c'**, **2'**, and **3'** (60-40 ppm, molecular structure and carbon labelling of the precursors are reported in Figure S 3)[16–18].

The spectrum of the copolymer PH is dominated by the resonance of -OCH_2- (**4**) of PEGDA main chain at 71 ppm, and contains all signals of the two components. Notably, the C=O resonance (**a**, **1**) shows a chemical shift intermediate between those observed in PEGDA and HEMA, suggesting effective copolymerization. In the 60–40 ppm region, methylene signals attributable to copolymerization are also observed.

In the PHD sample, the appearance of new resonances and an overall peak sharpening are observed. DTT is recognised by the peaks at 72.5 and 35.7 ppm. The downfield shift of the latter (**I"**) indicates complete reaction with the copolymer[19], further supported by the upfield shift of **3'** to 28 ppm (**3"**) due to the formation of a methylene-sulfur bond. The sharpening of other resonances, such as **1** and **4,** relatively close to the -CH_2-S bond, suggests an increase in the molecular mobility of the PH copolymer. A preferential bond between DTT and PEGDA rather than with HEMA can be assumed, as the broad signal at ~42 ppm (assigned to **2'** and **3'** of PEGDA) disappears, while the HEMA **c'** peak remains.

These spectral features are retained in both final formulations, PHDB and PHDBEV, obtained by mixing the polymeric precursors with the DB complex. In these spectra, the resonance at ~77 ppm is assigned to borax-complexed DTT (**II'**), and the small downfield shoulders of **I"** and **3"**, at 37.6 and 29.3 ppm indicate the sensitivity of these functional groups to the proximity to C–O–B bond; indeed, the intensities of the original **I"** and **3"** peaks decrease relative to those in the PHD sample, as a proof of the signal splitting. All these features prove that DB complex exists and effectively forms sulphur bridges with the copolymer. Furthermore, the asymmetric lineshapes suggest the possible presence of different complexes, such as 1:1 and 2:1 complex.

The ^{11}B MAS NMR spectra of the PHDB and PHDBEV samples after swelling in water (20 μL in 100 mg, to reduce anisotropy and improving resolution) are similar. PHDB shows a broad resonance centred at ~14 ppm, corresponding to $B(OH)_3/B(OH)_4$[illegible] species in dynamic exchange on the NMR timescale (the rapid exchange on boron leads to a broad resonance signal instead of two distinct peaks, because the ^{11}B nucleus is experiencing a changing environment), a sharp peak at 9.6 ppm from a di-chelate complex, and a minor sharp peak at 5.1 ppm, attributable to a 1:1 complex[1,15,20,21].

The PHDBEV spectrum appears largely similar. The peaks corresponding to the 1:1 and 2:1 complexes remain unchanged, while the broad signal shifts downfield to 18 ppm, consistent with its sensitivity to surrounding matrix properties[20,22]. A new small sharp resonance appears at 11.2 ppm. The assignment of this peak is not straightforward. According to Coddington, it could originate from triborate ions[15]; alternatively, Valenzuela et al. attributed similar minor resonances to solvent adducts[22]. Given the abundance of hydroxyl groups in the molecular structure of the tannin extract Extratan Vinacciolo, adduct formation seems plausible.

Finally, it is worth noting that the presence of both mono-chelate and di-chelate complexes supports the hypotheses proposed in the discussion of the ^{13}C spectra.

FTIR

Figure S4a shows the FTIR spectra of DTT, borax and of the DTT+Borax complex (DB). DTT's spectrum showed characteristic absorption peaks for hydroxyl groups (broad doublet around 3300 cm^{-1}), aliphatic C–H stretching (2970–2910 cm^{-1}), and thiol (S–H) stretching at 2570–2580 cm^{-1}. Peaks at 1410 cm^{-1} and 1230–1320 cm^{-1} were assigned to CH_2 bending and scissoring modes, while bands at 1050 and 1100 cm^{-1} corresponded to C–O stretching vibrations. A C–S stretch was also identified at 675 cm^{-1} [23]. In the borax spectrum, broad absorptions around 3400 and 1670 cm^{-1} were associated with O–H stretching and H–O–H bending, respectively. Distinctive bands at 1350, 1120, 1065, 940, 830, and 705 cm^{-1} were attributed to B–O stretching vibrations in BO_3 and BO_4 units, in agreement with literature data [24,25].

Upon mixing DTT and borax, the FTIR spectrum revealed new features indicative of borate–ester formation. A strong band appeared in the 1300–1400 cm^{-1} range, commonly associated with asymmetric B–O–C stretching in borate esters. Additional

changes were observed between 1220–1260 cm^{-1} (C–O stretches), 1000–1060 cm^{-1} (B–O stretches), and 550–700 cm^{-1} (out-of-plane B–O vibrations), consistent with previous reports [26,27].

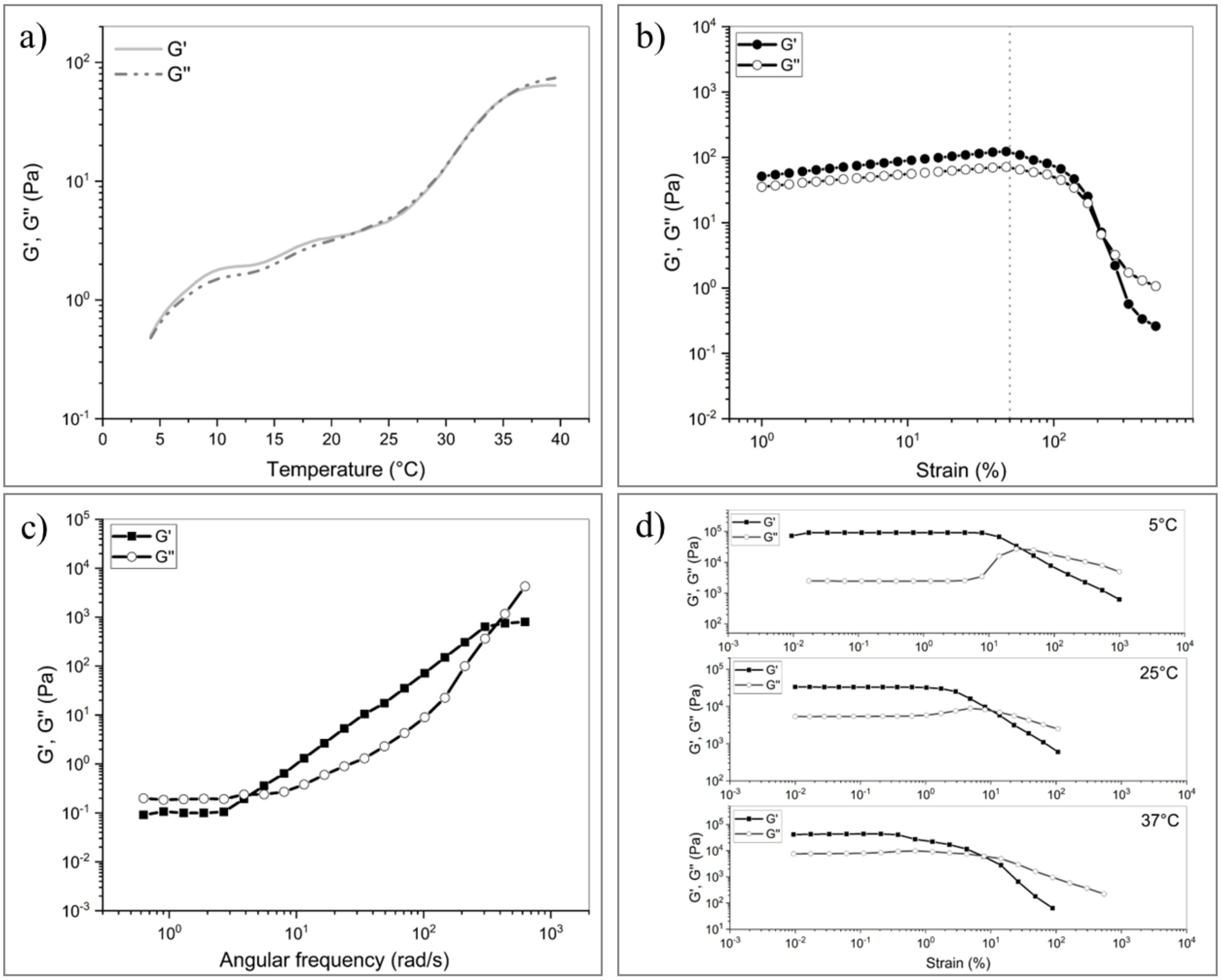

Figure S 2 – (**a**) Temperature ramp on the liquid formulation: values of G' and G'' depending on temperature; (**b**) amplitude sweep on the liquid formulation; (**c**) frequency sweep on the liquid formulation; (**d**) amplitude sweep on the gelled samples conducted at 5, 25 and 37°C . All tests conducted at room temperature unless specified.

The polymeric network was further analyzed by comparing FTIR spectra of polymeric precursors PEGDA and HEMA and of the three hydrogel formulations: (i) PEGDA+HEMA (PH), (ii) PEGDA+HEMA+DTT (PHD), and (iii) PEGDA+HEMA+DTT+borax (PHDB), all prepared in PBS with LAP photoinitiator and dried post-curing. Spectra were normalized to the ester carbonyl band at 1720 cm^{-1} [28]. Results are shown in Figure S3b. In PH, absorption bands for C=O (1720 cm^{-1}), C=C (1633 cm^{-1}), CH_2/CH_3 stretches (2870–2945 cm^{-1}), and OH groups (3200–3600 cm^{-1}) were observed. A minor signal at 1650 cm^{-1} indicated residual unreacted HEMA [29]. Upon addition of DTT (PHD), no substantial spectral shifts were noted except for minor enhancements in OH and C–O regions.

In the fully crosslinked hydrogel (PHDB), two key features emerged: (i) a marked increase in the 1350–1450 cm^{-1} region, corresponding to B–O–C stretches of borate–ester bonds [30]; and (ii) distinct peaks at 705 and 680 cm^{-1}, attributed to B–O (out-of-plane) and C–S vibrations, respectively [23,24]. These spectral changes confirm the integration of DTT and borax through dynamic covalent borate–diol interactions, consistent with the crosslinking mechanism proposed.

Table S 1. Viscosity values at different shear rates and temperatures

Temperature (°C)	Viscosity (Pa·s) (γ=1s^{-1})	Viscosity (Pa·s) (γ=1000s^{-1})
**5**	0.032	0.022
**15**	0.022	0.016
**25**	0.015	0.009
**35**	0.017	0.009

Table S 2. Photo-rheological parameters

Latency time (s)	Gel point (s)	Total reaction time (s)*	Final G' (kPa)	Cross-link density (m^{-3})
7	15	140	122	$2.96 \cdot 10^{25}$

* time at which 80% of the final G' is reached

Table S 3. Composition and labeling of reference samples for NMR and FTIR spectroscopic analysis

Label	Composition	
**PEGDA**	Polyethylenglycol diacrylate	+1% LAP<br>3D printed
**HEMA**	Hydroxyethyl methacrylate	+1% LAP<br>3D printed

**DTT**	Dithiotreithol	Powder
**Borax**	Sodium tetraborate	Powder
**DB**	DTT : Borax = 3 : 1	80 wt% PBS<br>Dried
**PH**	PEGDA : HEMA = 2 : 1	+1% LAP<br>80 wt% PBS<br>3D printed and then dried
**PHD**	DTT : copol = 1 : 2<br>PEGDA : HEMA = 2 : 1	+1% LAP<br>80 wt% PBS<br>3D printed and then dried
**PHDB**	DTT : copol = 1 : 2<br>DTT . Borax = 3 : 1<br>PEGDA : HEMA = 2 : 1	+1% LAP<br>80 wt% PBS<br>3D printed and then dried
**PHDBEV**	DTT : copol = 1 : 2<br>DTT . Borax = 3 : 1<br>PEGDA : HEMA = 2 : 1<br>Radical Scavenger 0.5phr	+1% LAP<br>80 wt% PBS<br>3D printed and then dried

Table S 4. ^{11}B MAS NMR profile fitting results on swollen samples.

δ (ppm)	17.5 + 0.5	11.5	9.8	5.5
	Free boron	DB-EV*	2:1 complex	1:1 complex
PHDBEV	68.3%	2.7%	26.0%	3.0%
PHDB	73.9%	ND	24.5%	1.6%
DB	49.9%	ND	23.7%	26.4%

*probable adduct DB- radical scavenger

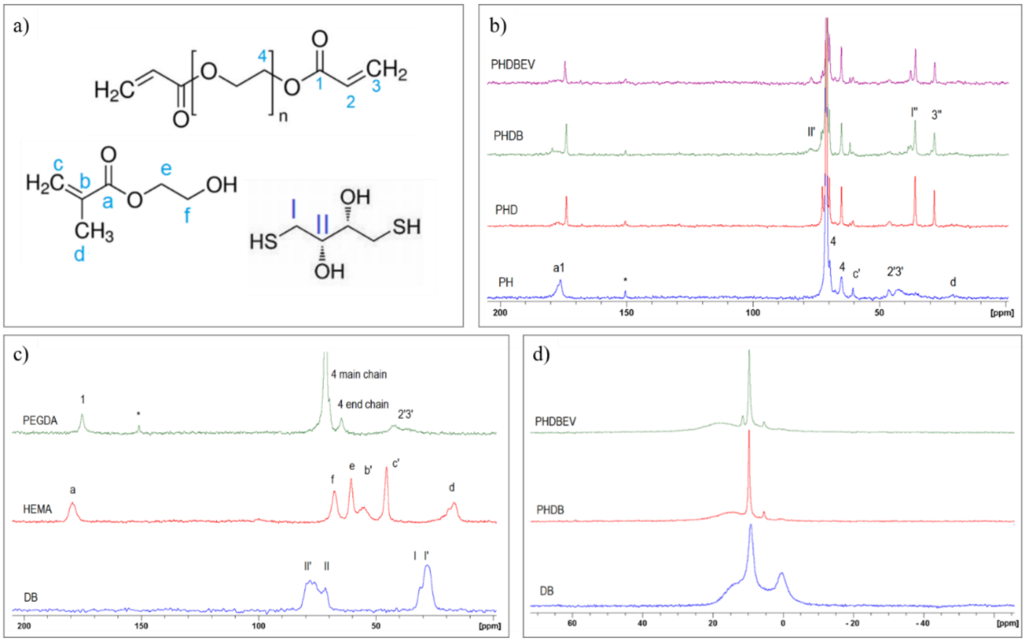

Figure S 3. (**a**) Precursor molecular structures with C labelling of DTT, PEGDA and HEMA; (**b**) ^{13}C MAS NMR spectra of PH, PHD, PHDB and PHDBEV samples; (**c**) ^{13}C MAS NMR spectra of PEGDA, HEMA and DTT-Borax complex. * is a ssb of 4. ; (**d**) ^{11}B MAS NMR of formulations containing borax.

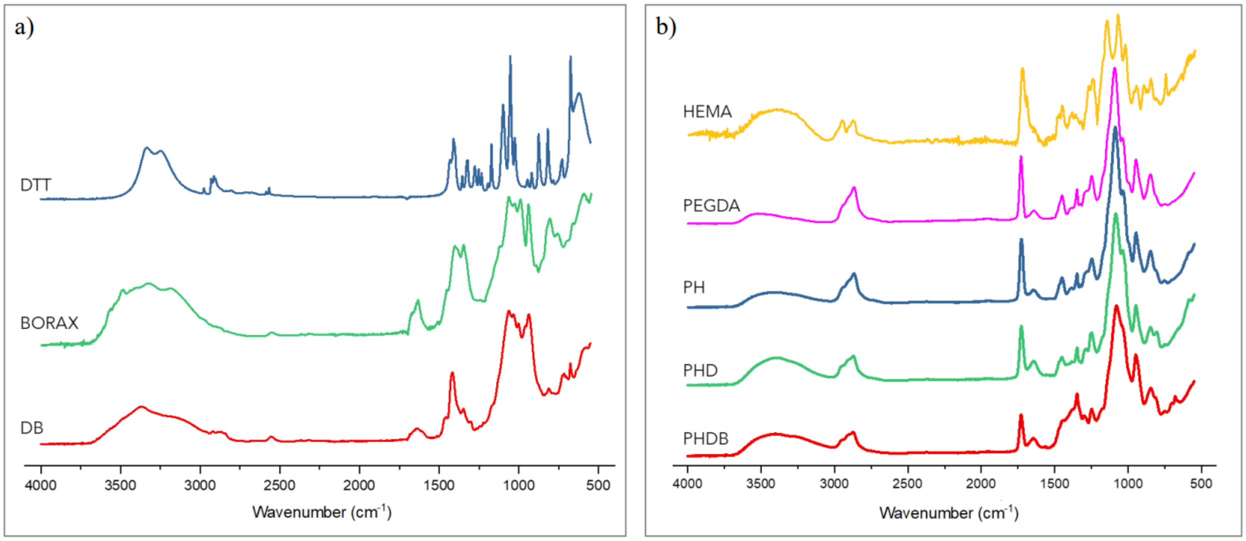

Figure S 4. (**a**) FTIR spectra of DTT, borax and DTT-Borax complex; (**b**) FTIR spectra of cured PEGDA and HEMA and of the hydrogels (PH, PHD, PHDB), normalized with respect to the 1720 cm^{-1} peak.

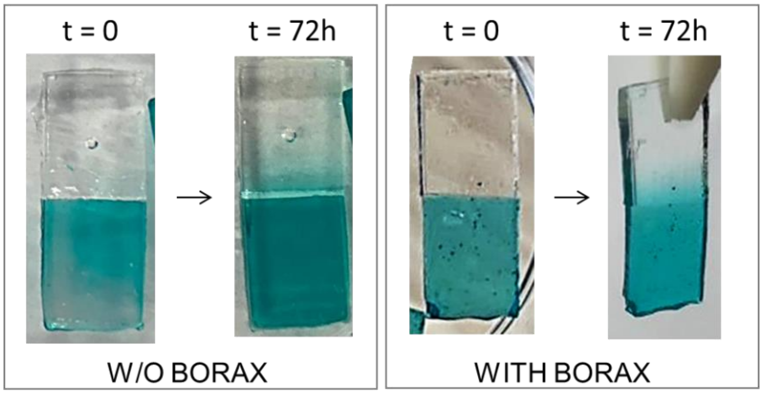

Figure S 5. Comparison of self-healing between samples with and without borax. Samples without borax show no signs of self-repair: the cut interface remained visible, the diffusion of the dye is minimal and the two halves failed to adhere or recover any mechanical continuity over time, while borax-containing samples underwent self-healing and are able to withstand their own weight and sustain tension. This demonstrates that SH is due to the presence of borax and its interaction with DTT.

Hydogel properties

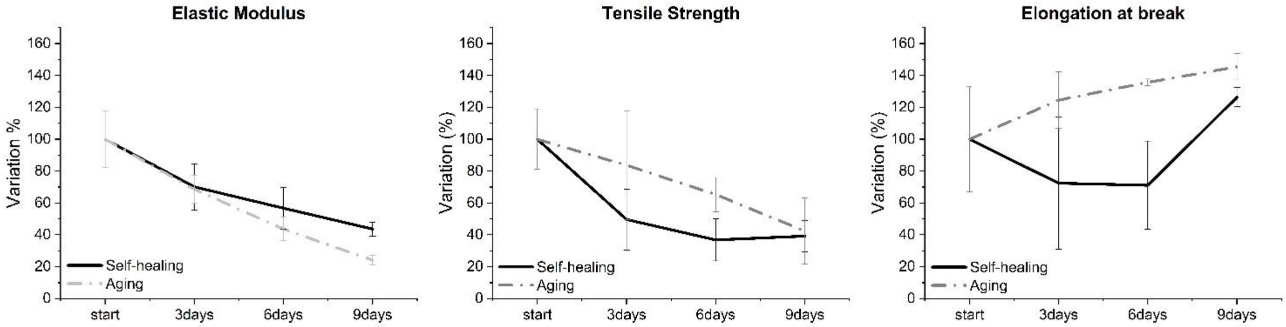

Figure S 6. Comparison between the effect of self-healing and aging on the variation of mechanical properties in tensile tests

Cytocompatibility

A viability test was performed using human lung adenocarcinoma cells (A549) cultured in medium previously conditioned by the hydrogel. The results, reported here, indicate a limited increase in cell number over time. Although the proliferative activity was low compared to the control, the cells remained viable, suggesting that no acute cytotoxic effect was induced by the conditioned medium. The modest cell growth observed could also be related to nutrient sequestration by the hydrogel during the conditioning phase, which may have reduced the availability of essential factors for cell proliferation.

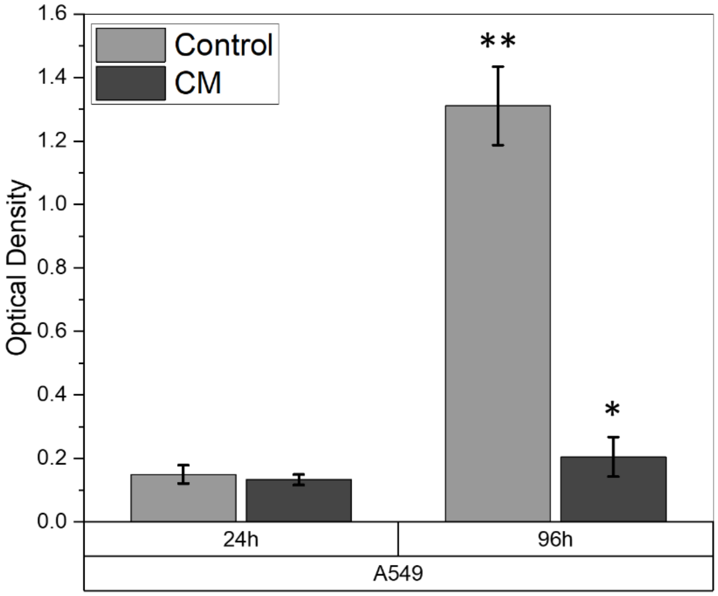

Figure S 7. MTT assay results on A549 cell lines after 24 and 96h (p-value *<0.05; **<0.01; ***<0.001).

Table S 5. Swelling degree in different solutions at 37°C

Time	PBS	pH5	pH7.4	pH9	H_2O	Gluc 0.5	Gluc 5	Gluc 50
0.5h	18 ± 4	20 ± 5	25 ± 3	35 ± 3.0	42.1 ± 2.8	37 ± 4	38 ± 9	38 ± 11
1h	31.9 ± 1.3	11 ± 4	41 ± 3	60.2 ± 1.9	63 ± 12	61 ± 3	61 ± 13	62 ± 14
1.5h	41 ± 3	13 ± 4	50 ± 5	77 ± 4	n.a.	76.1 ± 1.5	78 ± 11	77 ± 18
2h	45 ± 5	14 ± 3	54 ± 6	82 ± 10	75 ± 7	86.5 ± 1.9	90 ± 15	85 ±17
3h	52.7 ± 2.0	14 ± 3	56 ± 5	90 ± 10	82 ± 6	92.7 ± 0.6	97 ± 14	87 ± 16
18h	61 ± 6	13 ± 4	60 ± 6	124 ± 16	118 ± 5	105 ± 6	106 ± 13	88 ± 14
24h	65 ± 6	10 ± 4	63 ± 5	138 ± 8	129 ± 18	111 ± 9	114 ± 14	87 ± 15
4 days	86 ± 16	11 ± 5	97 ± 10	-57 ± 24	n.a.	157 ± 14	117 ± 16	70 ± 13
7 days	84 ± 14	0 ± 50	102 ± 30	n.a.	183 ± 25	180 ± 30	117 ± 17	43 ± 18
11 days	86 ± 19	-62 ± 17	95 ± 37	n.a.	n.a.	170 ± 23	116 ± 25	31 ± 28
14 days	89 ± 9	n.a.	90 ± 22	n.a.	189 ± 23	156 ± 24	91 ± 4	44 ± 14

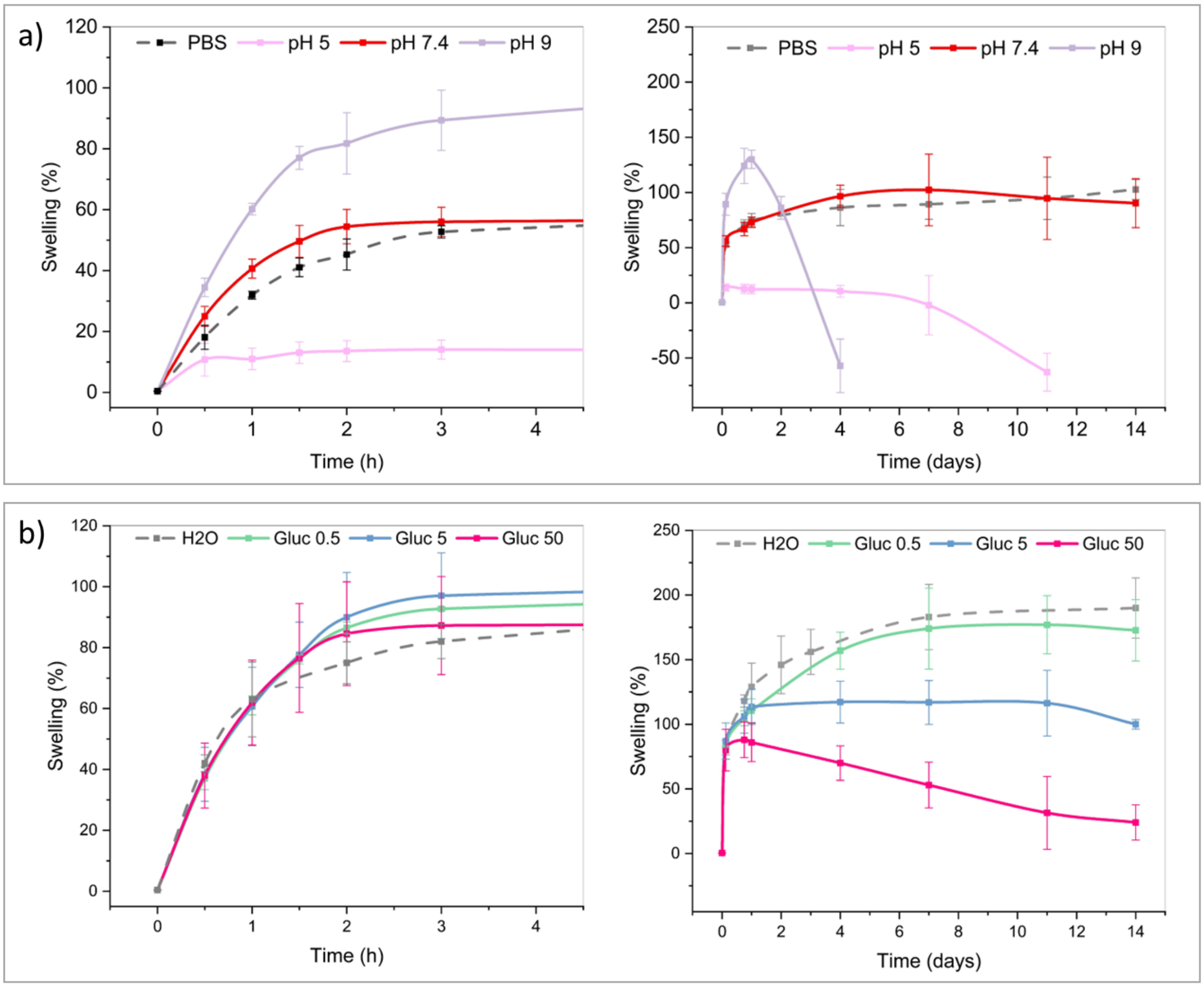

Figure S 8. Swelling behavior of hydrogels in solutions with varying (**a**) pH and (**b**) glucose concentrations over 14 days, with magnification of the first 4 hours on the left; PBS and deionized water are used as controls. In neutral media, hydrogels exhibit stable swelling (80–100%) without degradation, while in basic and acidic environments degradation occurs after approximately 3 and 7 days, respectively. In glucose-containing solutions, a higher swelling degree is observed compared to glucose-free controls, although the initial swelling rate appears largely independent of glucose concentration. In contrast, higher pH values result in a more rapid initial swelling.

References

1. He L, Szopinski D, Wu Y, Luinstra GA, Theato P. Toward Self-Healing Hydrogels Using One-Pot Thiol-Ene Click and Borax-Diol Chemistry. *ACS Macro Lett*. 2015;4(7):673-678. doi:10.1021/acsmacrolett.5b00336

2. Zhu G, Zhang J, Huang J, et al. High-performance 3D printing UV-curable resins derived from soybean oil and gallic acid. *Green Chemistry*. 2021;23(16). doi:10.1039/d1gc01934a

3. Rekowska N, Huling J, Brietzke A, et al. Thermal, Mechanical and Biocompatibility Analyses of Photochemically Polymerized PEGDA250 for Photopolymerization-Based Manufacturing Processes. *Pharmaceutics*. 2022;14(3). doi:10.3390/pharmaceutics14030628

4. Pradeep P V., Paul L. Review on novel biomaterials and innovative 3D printing techniques in biomedical applications. *Mater Today Proc*. 2022;58. doi:10.1016/j.matpr.2022.01.072

5. Choi JR, Yong KW, Choi JY, Cowie AC. Recent advances in photo-crosslinkable hydrogels for biomedical applications. *Biotechniques*. 2019;66(1). doi:10.2144/btn-2018-0083

6. McAvoy K, Jones D, Thakur RRS. Synthesis and Characterisation of Photocrosslinked poly(ethylene glycol) diacrylate Implants for Sustained Ocular Drug Delivery. *Pharm Res*. 2018;35(2). doi:10.1007/s11095-017-2298-9

7. Engebretson B, Sikavitsas VI. Long-term in vivo effect of peg bone tissue engineering scaffolds. *J Long Term Eff Med Implants*. 2012;22(3). doi:10.1615/JLongTermEffMedImplants.2013006244

8. Liu G, Li Y, Yang L, et al. Cytotoxicity study of polyethylene glycol derivatives. *RSC Adv*. 2017;7(30). doi:10.1039/c7ra00861a

9. O'Donnell K, Boyd A, Meenan BJ. Controlling fluid diffusion and release through mixed-molecular-weight poly(ethylene) glycol diacrylate (PEGDA) hydrogels. *Materials*. 2019;12(20). doi:10.3390/ma12203381

10. Veerubhotla K, Lee CH. Design of biodegradable 3D-printed cardiovascular stent. *Bioprinting*. 2022;26. doi:10.1016/j.bprint.2022.e00204

11. Macková H, Plichta Z, Hlídková H, et al. Reductively Degradable Poly(2-hydroxyethyl methacrylate) Hydrogels with Oriented Porosity for Tissue Engineering Applications. *ACS Appl Mater Interfaces*. 2017;9(12). doi:10.1021/acsami.7b01513

12. Passos MF, Carvalho NMS, Rodrigues AA, et al. PHEMA Hydrogels Obtained by Infrared Radiation for Cartilage Tissue Engineering. *International Journal of Chemical Engineering*. 2019;2019. doi:10.1155/2019/4249581

13. Kazemi Ashtiani M, Zandi M, Shokrollahi P, Ehsani M, Baharvand H. Surface modification of poly(2-hydroxyethyl methacrylate) hydrogel for contact lens application. *Polym Adv Technol*. 2018;29(4). doi:10.1002/pat.4233

14. Cocarta AI, Hobzova R, Trchova M, et al. 2-Hydroxyethyl Methacrylate Hydrogels for Local Drug Delivery: Study of Topotecan and Vincristine Sorption/Desorption Kinetics and

Polymer-Drug Interaction by ATR-FTIR Spectroscopy. *Macromol Chem Phys*. 2021;222(13). doi:10.1002/macp.202100086

15. Coddington JM, Taylor MJ. High field11B and 13C nmr investigations of aqueous borate solutions and borate-diol complexes. *J Coord Chem*. 1989;20(1). doi:10.1080/00958978909408845

16. Zellander A, Zhao C, Kotecha M, et al. Characterization of pore structure in biologically functional poly(2-hydroxyethyl methacrylate) - Poly(ethylene glycol) diacrylate (PHEMA-PEGDA). *PLoS One*. 2014;9(5). doi:10.1371/journal.pone.0096709

17. Yang X, Zhou L, Lv L, Zhao X, Hao L. Multi-stimuli-responsive poly(NIPA-co-HEMA-co-NVP) with spironaphthoxazine hydrogel for optical data storage application. *Colloid Polym Sci*. 2016;294(10). doi:10.1007/s00396-016-3915-6

18. Son KH, Lee JW. Synthesis and characterization of poly(ethylene glycol) based thermo-responsive hydrogels for cell sheet engineering. *Materials*. 2016;9(10). doi:10.3390/ma9100854

19. Laurano R, Cassino C, Ciardelli G, Chiono V, Boffito M. Polyurethane-based thiomers: A new multifunctional copolymer platform for biomedical applications. *React Funct Polym*. 2020;146. doi:10.1016/j.reactfunctpolym.2019.104413

20. Sinton SW. Complexation Chemistry of Sodium Borate with Poly(vinyl alcohol) and Small Diols: A11B NMR Study. *Macromolecules*. 1987;20(10). doi:10.1021/ma00176a018

21. Blue RM, Macho JM, Lee HW, Macmillan JB. 11B and 1H-11B HMBC NMR as a Tool for Identification of a Boron-Containing Nucleoside Dimer. *J Nat Prod*. 2022;85(11). doi:10.1021/acs.jnatprod.2c00745

22. Valenzuela SA, Howard JR, Park HM, Darbha S, Anslyn E V. 11B NMR Spectroscopy: Structural Analysis of the Acidity and Reactivity of Phenyl Boronic Acid-Diol Condensations. *Journal of Organic Chemistry*. 2022;87(22). doi:10.1021/acs.joc.2c01514

23. Nicolas J, Jaafar M, Sepetdjian E, et al. Redox activity and chemical interactions of metal oxide nano- and micro-particles with dithiothreitol (DTT). *Environmental Sciences: Processes and Impacts*. 2015;17(11). doi:10.1039/c5em00352k

24. Goel N, Sinha N, Kumar B. Growth and properties of sodium tetraborate decahydrate single crystals. *Mater Res Bull*. 2013;48(4). doi:10.1016/j.materresbull.2013.01.007

25. Gautam C, Yadav AK, Singh AK. A Review on Infrared Spectroscopy of Borate Glasses with Effects of Different Additives. *ISRN Ceramics*. 2012;2012. doi:10.5402/2012/428497

26. Smith MK, Northrop BH. Vibrational properties of boroxine anhydride and boronate ester materials: Model systems for the diagnostic characterization of covalent organic frameworks. *Chemistry of Materials*. 2014;26(12). doi:10.1021/cm5013679

27. He L, Szopinski D, Wu Y, Luinstra GA, Theato P. Toward Self-Healing Hydrogels Using One-Pot Thiol-Ene Click and Borax-Diol Chemistry. *ACS Macro Lett*. 2015;4(7). doi:10.1021/acsmacrolett.5b00336

28. Imani M, Sharifi S, Mirzadeh H, Ziaee F. Monitoring of polyethylene glycoldiacrylate-based hydrogel formation by real time NMR spectroscopy. *Iranian Polymer Journal (English Edition)*. 2007;16(1).

29. Passos MF, Dias DRC, Bastos GNT, et al. pHEMA hydrogels: Synthesis, kinetics and in vitro tests. *J Therm Anal Calorim*. 2016;125(1). doi:10.1007/s10973-016-5329-6

30. Larkin P. *Infrared and Raman Spectroscopy: Principles and Spectral Interpretation*. 2011. doi:10.1016/C2010-0-68479-3
